# Genomic evidence for genetic erosion and strong population subdivision in *Amomum hainanense* (Zingiberaceae), an endangered island endemic plant from Hainan, China

**DOI:** 10.1093/aobpla/plag045

**Published:** 2026-09-24

**Authors:** Liang Tang, Xu Wu, Yi-ting Xu, Lang-xing Yuan, Lian Zhou, Shou-bai Liu

**Affiliations:** Hainan Baoting Tropical Forest Ecosystem Observation and Research Station, School of Ecology, Hainan University, 58 Renmin Avenue, Haikou 570228, Hainan, China; International Joint Center for Terrestrial Biodiversity Around South China Sea of Hainan Province, School of Ecology, Hainan University, 58 Renmin Avenue, Haikou 570228, Hainan, China; Hainan Baoting Tropical Forest Ecosystem Observation and Research Station, School of Ecology, Hainan University, 58 Renmin Avenue, Haikou 570228, Hainan, China; Hainan Baoting Tropical Forest Ecosystem Observation and Research Station, School of Ecology, Hainan University, 58 Renmin Avenue, Haikou 570228, Hainan, China; Tropical Crops Genetic Resources Institute, Chinese Academy of Tropical Agricultural Sciences, No. 4 Xueyuan Road, Longhua District, Haikou 571101, Hainan, China; State Key Laboratory of Systematic and Evolutionary Botany, Institute of Botany, Chinese Academy of Sciences, No. 20 Nanxincun, Xiangshan, Beijing 100093, China; School of Tropical Agriculture and Forestry, Hainan University, 58 Renmin Avenue, Haikou 570228, Hainan, China

**Keywords:** ddRAD-seq, population structure, demographic history, conservation units, plastome phylogenomics, Hainan Island

## Abstract

*Amomum hainanense* is an endangered understorey herb narrowly distributed in tropical montane forests of Hainan Island, China, but its evolutionary history and conservation status remain poorly understood because genomic information is scarce. Here, we used double-digest restriction site-associated DNA sequencing to assess genetic diversity, population structure and demographic history in *Amomum hainanense*, and assembled complete chloroplast genomes from low-coverage whole-genome sequencing reads to clarify its phylogenetic placement. Genome-wide single nucleotide polymorphisms revealed exceptionally low nucleotide diversity (*π* = 0.7–1.7 × 10^−4^ among the five well-sampled populations; mean = 1.2 × 10^−4^), substantially lower than values reported for cultivated source taxa of Amomi Fructus (4.1–10.4 × 10^−4^). Pronounced population structure and high, statistically significant pairwise genetic differentiation (*F*_ST_ = 0.280–0.621) were consistent with restricted connectivity among populations. Stairway Plot 2 based on both pooled and principal component analysis-defined group-level folded site frequency spectra recovered a broadly consistent long-term decline in effective population size over the last ca. 10 000 years. Chloroplast genomes showed very low intraspecific diversity (*π* ≈ 0.2 × 10^−4^) and recovered *A. hainanense* as a strongly supported monophyletic lineage. Phylogenetic analysis of three plastid loci further identified *A. repoeense* as the closest sampled relative. Together, these results indicate severe genetic erosion coupled with strong population subdivision in *A. hainanense*. We recommend prioritizing *in situ* protection of the highest-diversity populations (XL and MLD) and establishing population-stratified *ex situ* germplasm collections that represent all major genetic groups.

## Teaser

Genomic data reveal that the endangered Hainan endemic *Amomum hainanense* has extremely low genetic diversity, strong genetic separation among populations and a long-term decline in population size. These patterns indicate substantial genetic erosion despite the species occurring in protected forest habitats. Conservation should therefore combine habitat protection with genetic monitoring, priority protection of the most diverse populations and population-stratified germplasm collections representing all major genetic groups.

## Introduction

Zingiberaceae is one of the largest monocot families, comprising more than 50 genera and over 1600 species, and reaches its highest species richness in tropical and subtropical Asia (Wu and Larsen 2000, Kress *et al.* 2002). Members of this family are perennial herbs with aromatic rhizomes, and many have substantial economic value as spices, medicines and ornamentals (Larsen *et al.* 1998, Pancharoen *et al.* 2000, Wu and Larsen 2000). Within Zingiberaceae, *Amomum* is among the most species-rich and economically important genera, largely because it includes medicinal and aromatic taxa (Cai *et al.* 2021, Imran *et al.* 2024). Several species are widely used in traditional medicine and as flavouring agents, including *A. villosum* (the source of Amomi Fructus, ‘Sharen’) (Gao *et al.* 2023) and *A. tsao-ko* (tsaoko cardamom) (Yang *et al.* 2008, 2022). Phytochemical studies indicate that *Amomum* species are rich in volatile oils, terpenoids and flavonoids that contribute to antimicrobial, anti-inflammatory and digestive activities (Yang *et al.* 2008, Cai *et al.* 2021, Feng *et al.* 2024). *Amomum* therefore represents an important genetic resource for the food, pharmaceutical and perfumery industries.

Recent genomic and multi-omics studies have begun to reveal the genetic basis of aromatic metabolite biosynthesis in *Amomum*. A chromosome-level genome of *A. tsao-ko*, combined with metabolomic and transcriptomic analyses, has elucidated pathways underlying the formation of characteristic flavour compounds during fruit development (Li *et al.* 2022). Similarly, a high-quality genome assembly of *A. villosa* var. *villosa* has enabled molecular authentication of geo-authentic (‘Daodi’) medicinal materials marketed as ‘Sharen’, and comparative analyses indicated higher expression of terpenoid-biosynthesis genes in this variety than in closely related taxa (Chen *et al.* 2023). These advances highlight the value of genome-scale data for both applied research and the conservation of economically important *Amomum* species.

In China, approximately 24 *Amomum* species have been recorded, and many are harvested as spices or medicinal plants (Wu and Larsen 2000). Two species, *A. hainanense* and *A. petaloideum*, were listed as second-class nationally protected wild plants in the 2021 List of National Key Protected Wild Plants (Lu *et al.* 2021). *Amomum hainanense* is a narrowly distributed understorey herb of montane forests on Hainan Island (Fig. 1). It persists in small, geographically isolated populations that are likely vulnerable to genetic erosion and population decline (Zou *et al.* 2018). Nevertheless, genetic information for *A. hainanense* remains scarce, impeding robust assessments of genetic diversity, demographic history and conservation priorities.

**Figure 1:**
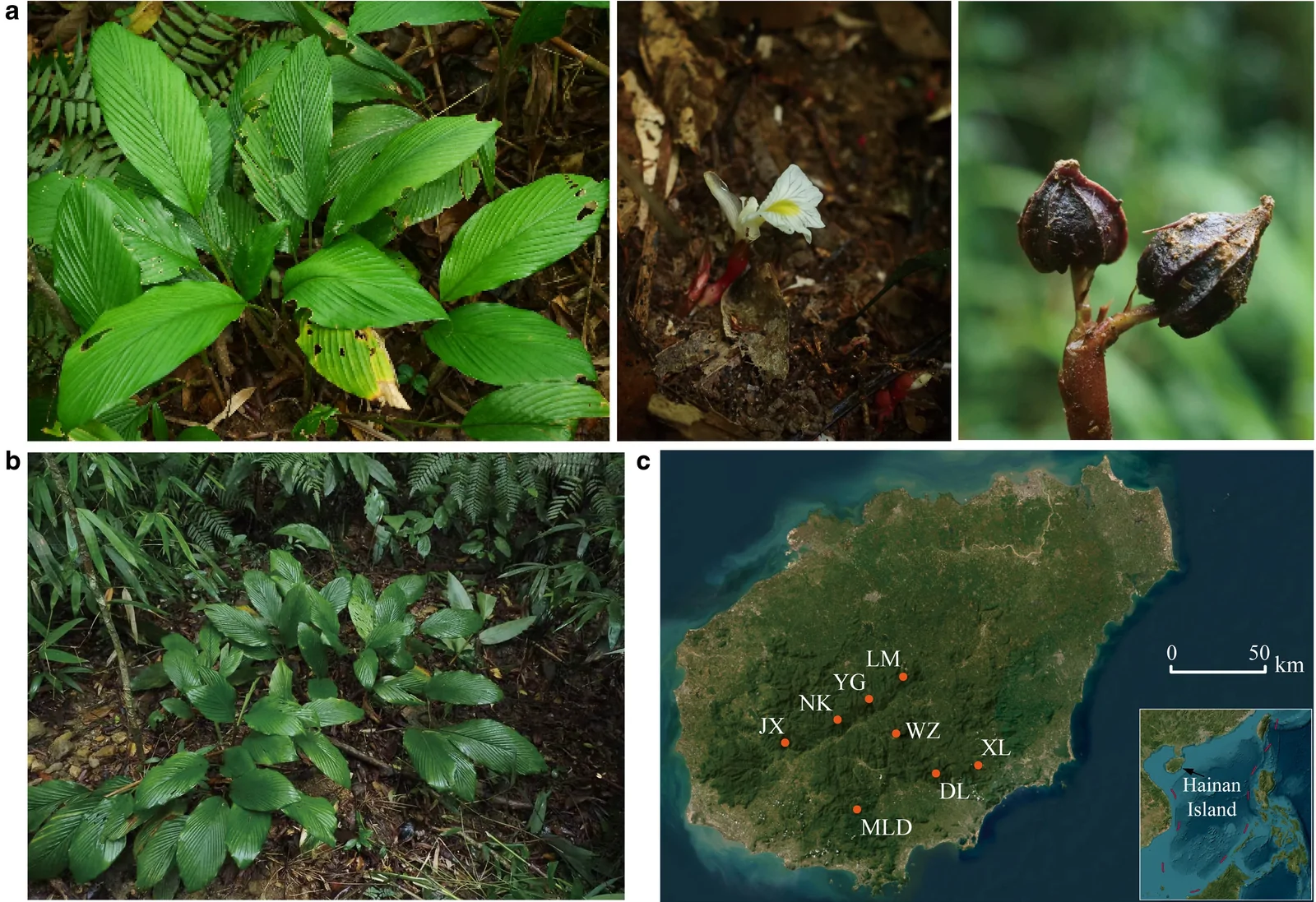
Study system of *Amomum hainanense*. (a) Morphology of *A. hainanense*, showing leafy shoots, flowers and fruits. (b) Representative understorey habitat of *A. hainanense* in montane forest on Hainan Island. (c) Geographic distribution of sampled populations on Hainan Island, China. Population abbreviations correspond to those in Table 1; dots indicate sampling localities. The inset shows the location of Hainan Island.

Conservation genetics provides a framework for diagnosing genetic risks in threatened species, delineating conservation units and guiding *in situ* and *ex situ* management (Frankham *et al.* 2010, Funk *et al.* 2012). Reduced-representation genome sequencing approaches, particularly restriction site-associated DNA sequencing (RAD-seq) and its derivatives [e.g. double-digest RAD-seq (ddRAD-seq)], enable efficient discovery of genome-wide single nucleotide polymorphisms (SNPs) in non-model plants that lack reference genomes (Davey and Blaxter 2010, Andrews *et al.* 2016). RAD-seq has been widely applied to threatened plants, such as the critically endangered aquatic plant *Caldesia grandis* (Yan *et al.* 2024), the rare recretohalophyte *Reaumuria trigyna* (Dang *et al.* 2024), endangered dipterocarps (*Hopea hainanensis* and *H. reticulata*) (Tang *et al.* 2024a, 2024b), the threatened tree peony *Paeonia ludlowii* (Zhao *et al.* 2023), and the medicinal herb *Salvia miltiorrhiza* and its relatives (Zhou *et al.* 2021). Collectively, these studies demonstrate the utility of RAD-derived SNP datasets for genome-wide conservation genetic assessment in threatened non-model plants.

Endangered island endemic plants represent particularly informative systems for conservation genomics because restricted ranges, small population sizes and limited gene flow can accelerate genetic erosion (Frankham 1997, Caujape-Castells *et al.* 2010). Genome-wide studies of endangered island endemic plants have shown that such species may harbour reduced genetic diversity and accumulate deleterious variation compared with widespread congeners (Hamabata *et al.* 2019). Studies of endangered plant lineages on oceanic archipelagos have further shown that high-throughput SNP data can reveal cryptic genetic structure and inform population management, especially when restoration requires decisions about seed sourcing, population mixing and *ex situ* collections (Gramazio *et al.* 2020). Although Hainan is a continental island rather than a remote oceanic archipelago, *A. hainanense* provides a comparable conservation-genomic case because it is a narrow-range island endemic restricted to small, isolated forest populations. For such species, a central conservation question is whether habitat protection alone is sufficient to maintain genetic diversity, or whether small remnant populations still require explicit genetic monitoring and population-level management.

In this study, we used ddRAD-seq to generate genome-wide nuclear SNPs for *A. hainanense* and used these data to assess its genetic diversity, population structure and differentiation, and demographic history. We also assembled complete chloroplast genomes from low-coverage whole-genome sequencing reads and used the plastome sequences, together with three plastid loci, to clarify the phylogenetic placement of *A. hainanense*. Overall, our results provide a genomic foundation for understanding the conservation genetic status of this endangered island endemic and for prioritizing conservation units and management actions.

## Methods

### Population sampling

A total of 69 individuals of *A. hainanense* were collected from eight montane localities across Hainan Island (Fig. 1). Five populations were represented by moderate sample sizes (*n* = 7–15 individuals per population), whereas three additional localities were represented by one to three individuals. Population sizes and geographic coordinates are provided in Table 1. Because many Zingiberaceae species form rhizomatous clumps, only one ramet was sampled per clump, and sampling points were spaced at least 5 m apart to minimize repeated sampling of the same genet. Mature, disease-free leaves were collected, rapidly dried in silica gel and stored at −20°C until DNA extraction.

**Table 1:** Sampling information and nucleotide diversity estimates for the 8 sampled localities of *Amomum hainanense* on Hainan Island.

Collection locality	Sample size	*π* × 10^–4^ (95% CI)	*H*_O_ (95% CI)	*H*_E_ (95% CI)	*F*_IS_ (95% CI)	Geographic coordinates	
						Longitude (°E)	Latitude (°N)
Diaoluo Shan (DL)	15	0.90 (0.87–0.93)	0.03621 (0.03331–0.03911)	0.08822 (0.08493–0.09151)	0.18907 (0.16702–0.21112)	109.871389	18.725833
Wuzhi Shan (WZ)	15	0.70 (0.67–0.73)	0.03411 (0.03101–0.03721)	0.05760 (0.05476–0.06044)	0.10111 (0.07830–0.12392)	109.680556	18.906111
Limu Shan (LM)	15	1.00 (0.97–1.03)	0.04251 (0.03932–0.04570)	0.08983 (0.08703–0.09263)	0.27312 (0.25031–0.29593)	109.714643	19.162213
Xinlong (XL)	12	1.60 (1.56–1.64)	0.04121 (0.03819–0.04423)	0.15419 (0.15019–0.15819)	0.35358 (0.33765–0.36951)	110.073747	18.762563
Maoladong reservoir (MLD)	7	1.70 (1.65–1.75)	0.03909 (0.03578–0.04240)	0.14567 (0.14126–0.15008)	0.31778 (0.30582–0.32974)	109.49365	18.563077
Jiaxi (JX)	3	0.40 (0.37–0.43)	0.03659 (0.03302–0.04016)	0.02690 (0.02457–0.02923)	NA	109.149145	18.864757
Nankai (NK)	1	0.70 (0.65–0.75)	0.05068 (0.04603–0.05533)	0.02534 (0.02303–0.02765)	NA	109.400383	18.968687
Yingge Ling (YG)	1	0.60 (0.56–0.64)	0.04024 (0.03603–0.04445)	0.02012 (0.01800–0.02224)	NA	109.550834	19.062064

Values in parentheses are 95% confidence intervals. Nucleotide diversity (π), observed heterozygosity (*H*_O_) and expected heterozygosity (*H*_E_) estimates for JX, NK and YG should be interpreted cautiously due to their small sample sizes. *F*_IS_ was not reported for the three localities because their sample sizes were too small for reliable estimation of within-population inbreeding coefficients. NA, not available because sample size is too small for reliable estimation.

### ddRAD-seq library construction, sequencing and SNP calling

Genomic DNA was extracted from silica-dried leaves using a Qiagen plant genomic DNA extraction kit (Qiagen, Shanghai, China). DNA quantity and quality were assessed using a Qubit 3.0 fluorometer. DNA samples were digested with EcoRI and MseI, followed by purification and size selection on agarose gel. The resulting fragments were ligated to barcoded adapters for individual identification. Libraries were PCR-enriched and sequenced on an Illumina NovaSeq X Plus platform in paired-end 150 bp mode.

Demultiplexing, RAD locus assembly, genotype calling and SNP filtering were conducted using Stacks v2 (Rochette *et al.* 2019). To optimize the *de novo* assembly of RAD loci, we tested the *ustacks* parameter *M* from 1 to 6 (*M*: maximum mismatches allowed between alleles within individuals) following recommendations for parameter optimization in *de novo* RAD locus assembly (Paris *et al.* 2017, Rochette and Catchen 2017). For each value of *M*, the catalog-building parameter *n* was set to the same value as *M*. We then ran the Stacks pipeline and recorded the number of retained RAD loci, polymorphic RAD loci and SNPs after filtering. The final analyses were conducted using *M* = 1 because this setting retained the largest number of polymorphic RAD loci while minimizing the risk of excessive merging of paralogous or highly divergent loci. The optimization results are provided in Supplementary Table S1. SNPs were retained if they met the following thresholds: (i) presence in all populations and in at least 80% of individuals per population; (ii) minor allele frequency ≥ 0.05; and (iii) maximum observed heterozygosity ≤ 0.7. SNPs with maximum observed heterozygosity > 0.7 were removed because unusually high heterozygosity in RAD-seq datasets may indicate collapsed paralogous loci, repetitive regions, erroneous locus merging or genotyping artefacts (O'Leary *et al.* 2018). This filtering step was therefore used to reduce the influence of potentially unreliable loci on downstream population genetic analyses. For population structure and demographic analyses, the *--write-random-snp* option in the Stacks *populations* programme was used to retain one randomly selected SNP per RAD locus, thereby avoiding the inclusion of multiple, potentially linked SNPs from the same locus. Two VCF datasets were therefore exported for downstream analyses: one containing all SNPs retained after filtering and the other containing the randomly selected SNPs. Both VCF datasets and the associated analysis input files are provided as individual Supporting Information files.

### Population genetic analyses and demographic inference

Genetic diversity statistics, including nucleotide diversity (*π*), observed and expected heterozygosity (*H*_O_ and *H*_E_), and the inbreeding coefficient (*F*_IS_) were estimated using the *populations* programme. The 95% confidence intervals for these estimates were calculated as estimate ± 1.96 × SE using the standard errors reported by Stacks. *F*_IS_ was not reported for JX, NK and YG because their sample sizes were too small for reliable estimation of within-population inbreeding coefficients. In addition, the three localities were not included in the comparison of pairwise genetic differentiation (*F*_ST_) between populations, and the estimated diversity statistics for them should be treated with caution. However, all sampled individuals were retained in STRUCTURE, PCA and neighbour-joining analyses to visualize the overall distribution of genetic variation.

Population structure was evaluated using STRUCTURE v2.3.4 (Pritchard *et al.* 2000) under the admixture model with correlated allele frequencies (Falush *et al.* 2003). For each *K* (1–12), ten independent runs were performed with a burn-in of 100 000 iterations followed by 1 000 000 Markov chain Monte Carlo simulations. The most likely *K* was inferred using STRUCTURE Harvester (Earl and vonHoldt 2012) following the Δ*K* method (Evanno *et al.* 2005), and clustering results were visualized with StructureSelector (Li and Liu 2018). Principal component analysis (PCA) was conducted using GCTA (Yang *et al.* 2011), and the first two principal components were used for visualization. Pairwise genetic distances were computed with VCF2Dis (https://github.com/BGI-shenzhen/VCF2Dis), and a neighbour-joining tree was reconstructed using fneighbor in the PHYLIP package with 1000 bootstrap replicates (Rice *et al.* 2000). Pairwise *F*_ST_ values among the five relatively well-sampled populations were estimated using the *populations* programme in Stacks. The significance of pairwise population differentiation was assessed in Arlequin ver 3.5.2.2 (Excoffier and Lischer 2010) using 1000 permutations based on the same filtered SNP dataset.

A one-dimensional folded site frequency spectrum (SFS) was constructed using easySFS (Gutenkunst *et al.* 2009) and the vcf2sfs R package (Liu *et al.* 2018). To accommodate missing data, we applied the down-projection procedure implemented in easySFS to select an appropriate projection value. Demographic history was inferred using Stairway Plot 2, a model-free, SFS-based approach that requires no reference genome and is especially suitable for RAD-derived SNP datasets (Liu and Fu 2015, 2020). To convert time from generations to years, we used a substitution rate of 7.389 × 10^−10^ per site per year, estimated from node ages and sequence data for a clade comprising representative *Alpinia* and *Amomum* species reported in Li *et al.* (2023). Field observations indicate that *A. hainanense* typically reaches reproductive maturity in its third year. We therefore assumed a 3-year generation time and assessed the sensitivity of demographic inference to alternative values of two and four years. All individuals collected in this study were pooled and used for the primary Stairway Plot 2 analyses. Because strong population structure may bias SFS-based demographic inference when individuals from differentiated populations are treated as a single randomly mating population, we additionally constructed folded SFSs for the two PCA-defined multi-locality genetic groups and repeated the Stairway Plot 2 analyses to assess whether the inferred demographic trend was robust to population subdivision. These two groups corresponded to group II (LM and XL) and a subset of group III (DL, WZ and JX), as indicated by the PCA and neighbour-joining tree topology. The single-individual localities NK and YG were excluded from the group-level SFS analyses.

### Chloroplast genome assembly and phylogenetic analysis

Low-coverage whole-genome sequencing was performed for 22 accessions of *A. hainanense* randomly selected from the 69 sampled individuals. Complete chloroplast genomes were assembled using GetOrganelle with default parameters (Jin *et al.* 2020). Representative plastome sequences of *Alpinia* and *Amomum* were retrieved from published datasets (Li *et al.* 2020, 2023, Zhang *et al.* 2021) and combined with the 22 newly assembled *A. hainanense* plastomes to build the phylogenomic dataset. Sequence alignments were generated using MAFFT (Katoh *et al.* 2019) and manually adjusted. Maximum-likelihood (ML) phylogenies were inferred in RAxML v8 under the GTR+Γ model, with 1000 rapid bootstrap replicates. *Zingiber officinale* (NC_044775.1) and *Curcuma longa* (MK262732.1) were used as outgroups to root the inferred ML tree.

Zou *et al.* (2018) suggested that *A. hainanense* morphologically resembles *A. repoeense* and *A. plicatum*. However, in our GenBank search we could retrieve chloroplast sequence data only for *A. repoeense*, limited to three loci: *matK* (KY620243.1), *rbcL* (JF940868.1) and the *psbA-trnH* intergenic spacer (JN043969.1). Comparable plastid sequences were not available for *A. plicatum*. We aligned these three loci of *A. repoeense* with the corresponding sequences from all taxa included in the plastome ML analyses to generate a three-locus dataset. This dataset was aligned and analysed using the same procedures and settings as the whole-plastome dataset. Chloroplast sequence diversity within *A. hainanense* was estimated from the 22 complete plastome alignments using DnaSP v6 (Rozas *et al.* 2017).

## Results

### Nucleotide diversity in *Amomum hainanense*

After quality control, 2 935 998 clean read pairs were retained per individual on average [see Supplementary Table S2]. Raw sequencing reads generated in this study are available in the NCBI Sequence Read Archive (SRA; BioProject accession PRJNA1442385). Based on the parameter-optimization results, *M* = 1 was selected for RAD locus assembly. After filtering, 36 882 loci were retained, of which 8716 polymorphic RAD loci contained a total of 12 702 SNPs. This SNP dataset was used to evaluate nucleotide diversity of *A. hainanense*. Application of the *--write-random-snp* option in the *populations* programme generated a linkage-reduced SNP dataset for population structure and demographic analyses.

Genetic diversity was generally low across all sampled localities of *Amomum hainanense* (Table 1). Three localities (JX, NK and YG) were represented by one to three individuals and were therefore excluded from population-level comparisons. Among the five well-sampled populations, expected heterozygosity was lowest in WZ (*H*_E_ = 0.05760) and highest in XL and MLD (*H*_E_ = 0.15419 and 0.14567, respectively). Inbreeding coefficients were positive across the five populations (*F*_IS_ = 0.10111 to 0.35358, mean = 0.24693). Nucleotide diversity (*π*) ranged from 0.70 × 10^−4^ (WZ) to 1.70 × 10^−4^ (MLD), with an overall mean of 1.2 × 10^−4^ (Table 1). Based on the 22 aligned complete plastomes, species-level chloroplast nucleotide diversity in *A. hainanense* was 0.2 × 10^−4^.

### Population structure

Model-based STRUCTURE analyses identified *K* = 7 as the most likely number of clusters under the Δ*K* criterion (Fig. 2a). At *K* = 7, *A. hainanense* exhibited pronounced genetic subdivision across Hainan Island (Fig. 2b). DL, LM, MLD and WZ were each dominated by distinct ancestry components, and the three individuals from JX were assigned primarily to the WZ cluster. Several individuals showed mixed membership; for example, a subset of DL individuals contained a fraction of the WZ component, and individuals from NK and YG showed predominantly WZ ancestry with a minor MLD component. The XL population was dominated by a single component, with several individuals showing mixed membership. Clustering results at *K* = 6 and *K* = 8 were broadly consistent with *K* = 7 in maintaining DL, LM, MLD, WZ and XL as distinct population-specific clusters. At *K* = 8, the JX individuals separated from WZ and formed an additional cluster. Although the highest supported *K* was *K* = 7, this value was not interpreted as the exact number of biological populations because several sampling localities had very small sample sizes. Moreover, two well-sampled populations (XL and LM) were further divided into two main components, indicating fine-scale genetic partitioning within these populations (Fig. 2b).

**Figure 2:**
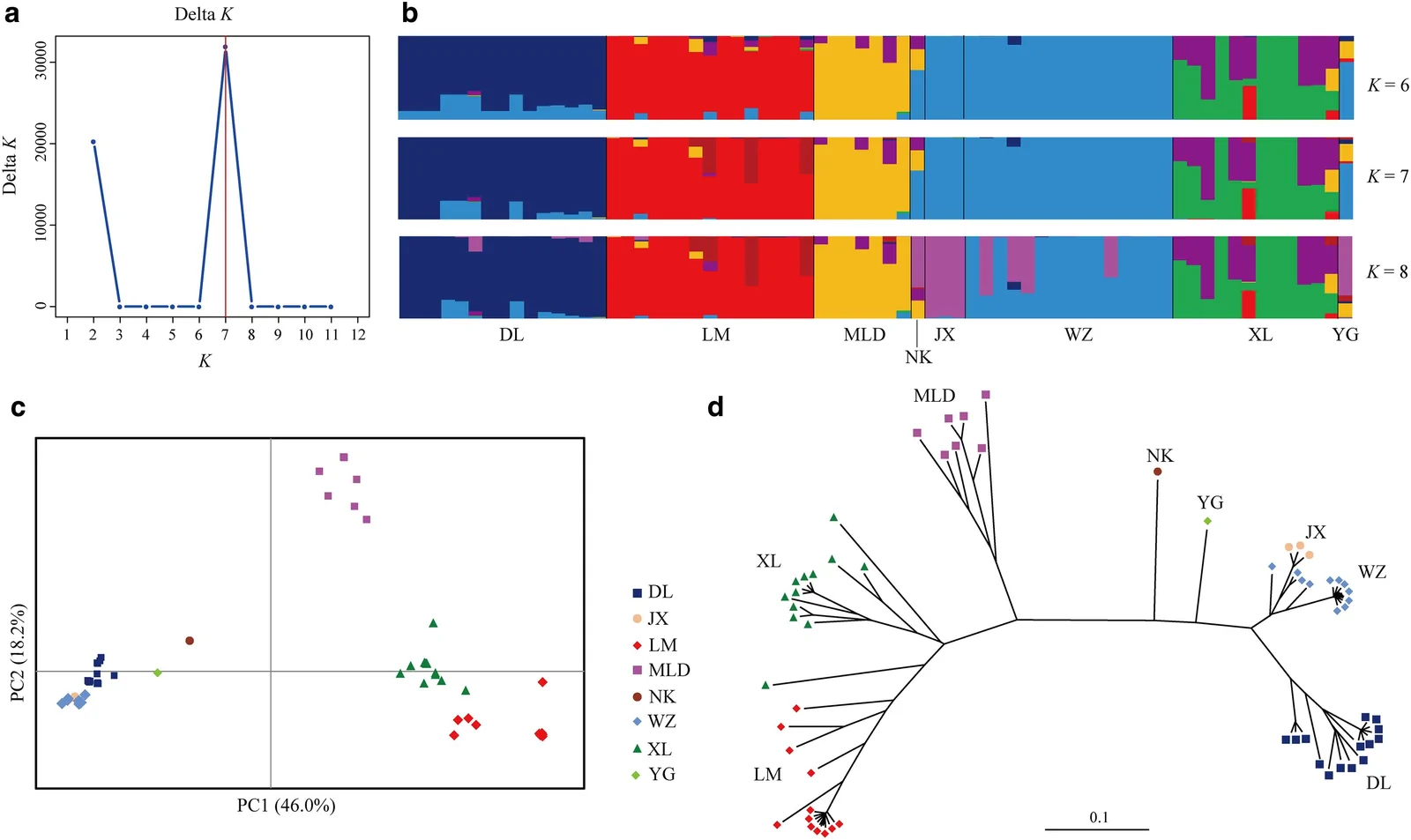
Population structure of *Amomum hainanense* inferred from ddRAD-seq SNPs. (a) Estimation of the most likely number of genetic clusters (*K*) using the Δ*K* method implemented in STRUCTURE Harvester; the red vertical line indicates *K* = 7. (b) STRUCTURE bar plots showing individual ancestry proportions at *K* = 6–8 under the admixture model; each vertical bar represents one individual and colours indicate inferred ancestry components. (c) Principal component analysis (PCA) of SNP genotypes showing the first two axes (PC1 = 46.0%, PC2 = 18.2%). (d) Neighbour-joining tree based on pairwise genetic distances among individuals. Tip colours and symbols correspond to sampled localities as shown in panel (c), and the scale bar indicates genetic distance.

The first two principal components explained 46.0% and 18.2% of the total genetic variation, respectively, and separated the sampled individuals into three major genetic groups (Fig. 2c): one corresponding to MLD alone (group I), a second comprising LM and XL (group II), and a third containing individuals from the remaining localities (DL, WZ, JX, NK and YG) (group III). The neighbour-joining tree broadly reflected the population differentiation detected by STRUCTURE and the major grouping pattern revealed by PCA (Fig. 2d). The three JX individuals overlapped with WZ in the PCA, consistent with their placement within WZ in the neighbour-joining tree. Because JX was represented by only three individuals, this locality was retained as a separate sampling locality but was not treated as an independent population-level unit. To reduce potential bias from very small sample sizes, JX, NK and YG were excluded from *F*_ST_ calculations. Pairwise *F*_ST_ among the five well-sampled populations ranged from 0.280 to 0.621 (mean = 0.457), confirming substantial population differentiation (Table 2). All pairwise comparisons were statistically significant based on Arlequin permutation tests (*P* < 0.001).

**Table 2:** Pairwise genetic differentiation (*F*_ST_) among populations of *Amomum hainanense*.

	DL	LM	MLD	WZ	XL
DL		<0.001	<0.001	<0.001	<0.001
LM	0.582830		<0.001	<0.001	<0.001
MLD	0.468227	0.450081		<0.001	<0.001
WZ	0.342341	0.621064	0.521213		<0.001
XL	0.438040	0.279634	0.369609	0.493885	

The lower triangular matrix contains estimated pairwise *F*_ST_ values, whereas the upper triangular matrix lists the corresponding permutation *P* values for these *F*_ST_ estimates.

### Demographic history

Stairway Plot 2 analyses based on folded site frequency spectra (SFSs) generated with easySFS and vcf2sfs using all individuals pooled consistently inferred a sustained reduction in effective population size (*N*_e_) over the last ca. 10 000 years (Fig. 3). The ancestral *N*_e_ before the decline was on the order of 3–7 × 10^4^ individuals, whereas contemporary *N*_e_ was substantially lower. Changes in generation time had only minor effects on the inferred timing of the contraction and estimates of ancestral effective population size [see Supplementary Figure S1]. Given the strong population subdivision detected in the SNP dataset, we further evaluated whether the inferred demographic trend was sensitive to the pooling of structured populations. Stairway Plot 2 analyses based on folded SFSs from two PCA-defined genetic groups recovered a broadly similar long-term decline in effective population size. Although the inferred timing and magnitude of *N*_e_ change differed to some extent among analyses, the overall declining trajectory was consistent between the pooled and group-based SFS analyses [see Supplementary Figure S2].

**Figure 3:**
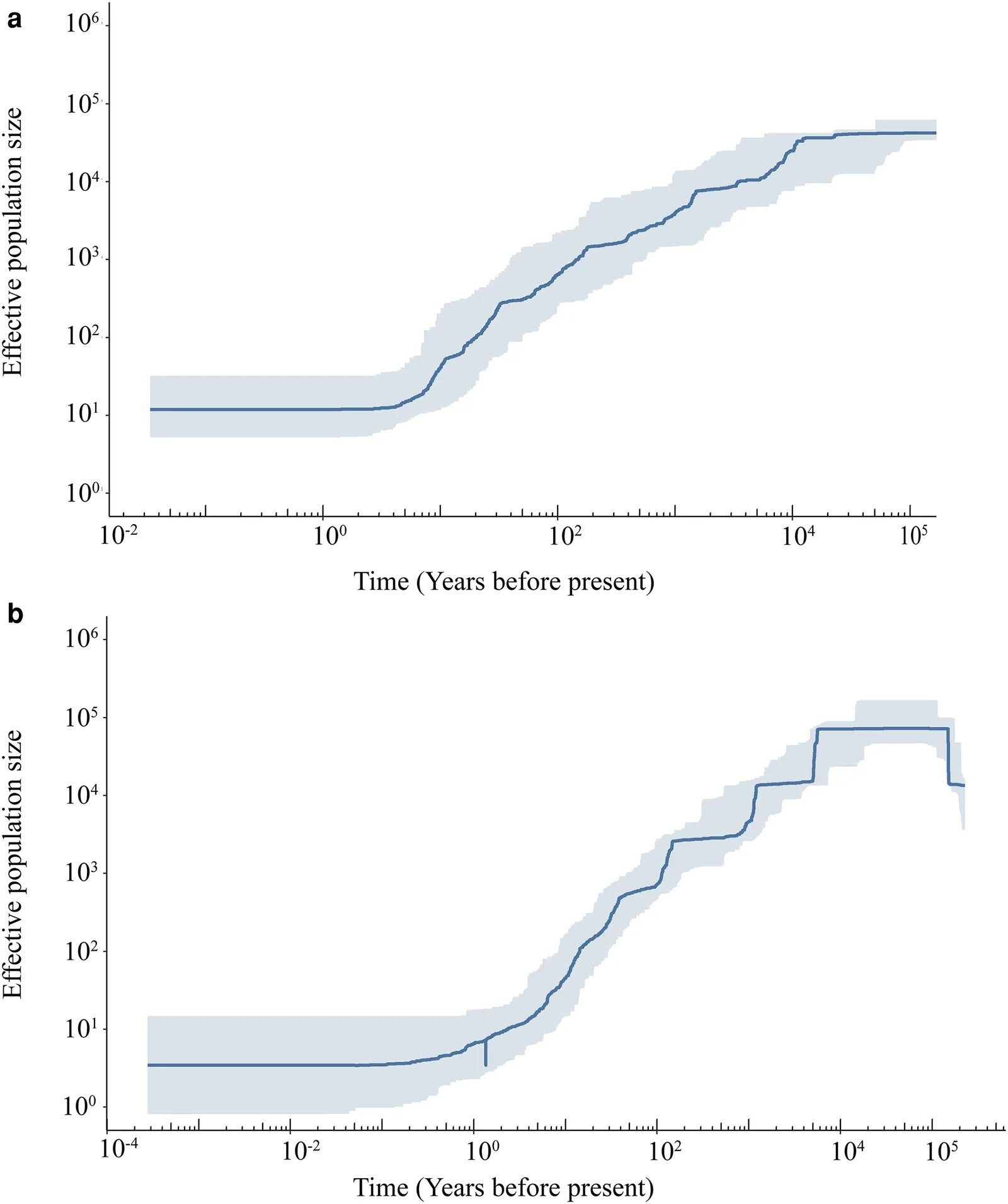
Demographic history of *Amomum hainanense* inferred using Stairway Plot 2 from folded site frequency spectra (SFSs) constructed with (a) easySFS and (b) vcf2sfs. Solid lines represent median estimates of effective population size (*N*_e_), and shaded areas indicate 95% confidence intervals.

### Chloroplast phylogenomics of *A. hainanense* and related taxa

An alignment of the 22 assembled complete plastomes of *A. hainanense* is provided as a Supporting Information file. The plastome alignment of *A. hainanense* and related taxa was 165 716 bp long and contained 5742 variable sites, of which 3144 were parsimony-informative. The ML phylogeny recovered *A. hainanense* as a strongly supported monophyletic clade (bootstrap support (BS) = 100), with very short internal and terminal branches, indicating minimal plastid divergence among sampled individuals.

The plastome ML tree resolved three major clades (Fig. 4). Clade I comprised a mixed assemblage of *Amomum* and *Alpinia* species, including *Amomum hainanense*, *A. maximum* and *A. longipetiolatum*, together with *Alpinia galanga* and *A. nigra*, whereas Clade II comprised only *Alpinia* species and Clade III only *Amomum* species in our sampling. Within Clade I, *A. maximum* and *A. longipetiolatum* formed a well-supported clade sister to *A. hainanense*, and all three *Amomum* species occur in montane regions of Hainan Island. Clade II comprised exclusively *Alpinia* species (*A. katsumadai*, *A. hainanensis*, *A. kwangsiensis*, *A. zerumbet*, *A. pumila*, *A. chinensis*, *A. officinarum* and *A. oxyphylla*), whereas Clade III contained only *Amomum* species (*A. villosum*, *A. longiligulare*, *A. neoaurantiacum*, *A. villosa*, *A. kravanh* and *A. compactum*). *Amomum tsao-ko* was recovered as sister to Clade I, albeit with low bootstrap support (BS = 62).

**Figure 4:**
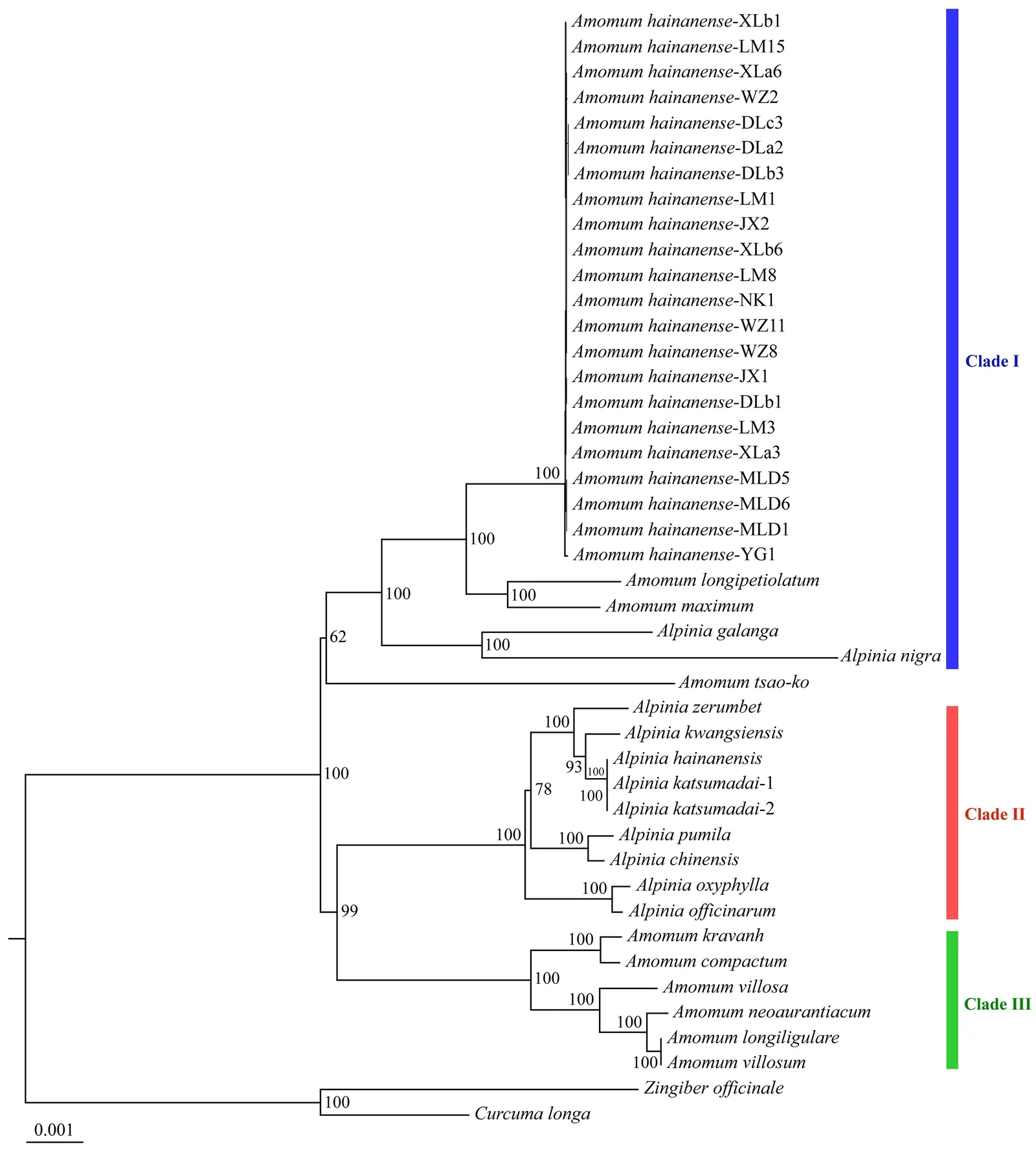
Maximum-likelihood (ML) phylogeny inferred from complete chloroplast genomes of 22 *A. hainanense* accessions and representative plastomes of *Amomum* and *Alpinia*. Numbers on branches are bootstrap support values (only key nodes shown). The scale bar indicates substitutions per site. Three major clades (I–III) are indicated on the right. *Zingiber officinale* and *Curcuma longa* were used as outgroups.

Because the 22 accessions of *A. hainanense* formed a well-supported monophyletic clade in the plastome ML phylogeny (Fig. 4), three representative accessions were retained for the three-locus analysis. The ML tree inferred from the three plastid loci placed *A. repoeense* as sister to *A. hainanense* (Fig. 5), and the overall topology and bootstrap support values were concordant with those from the plastome ML phylogeny.

**Figure 5.**
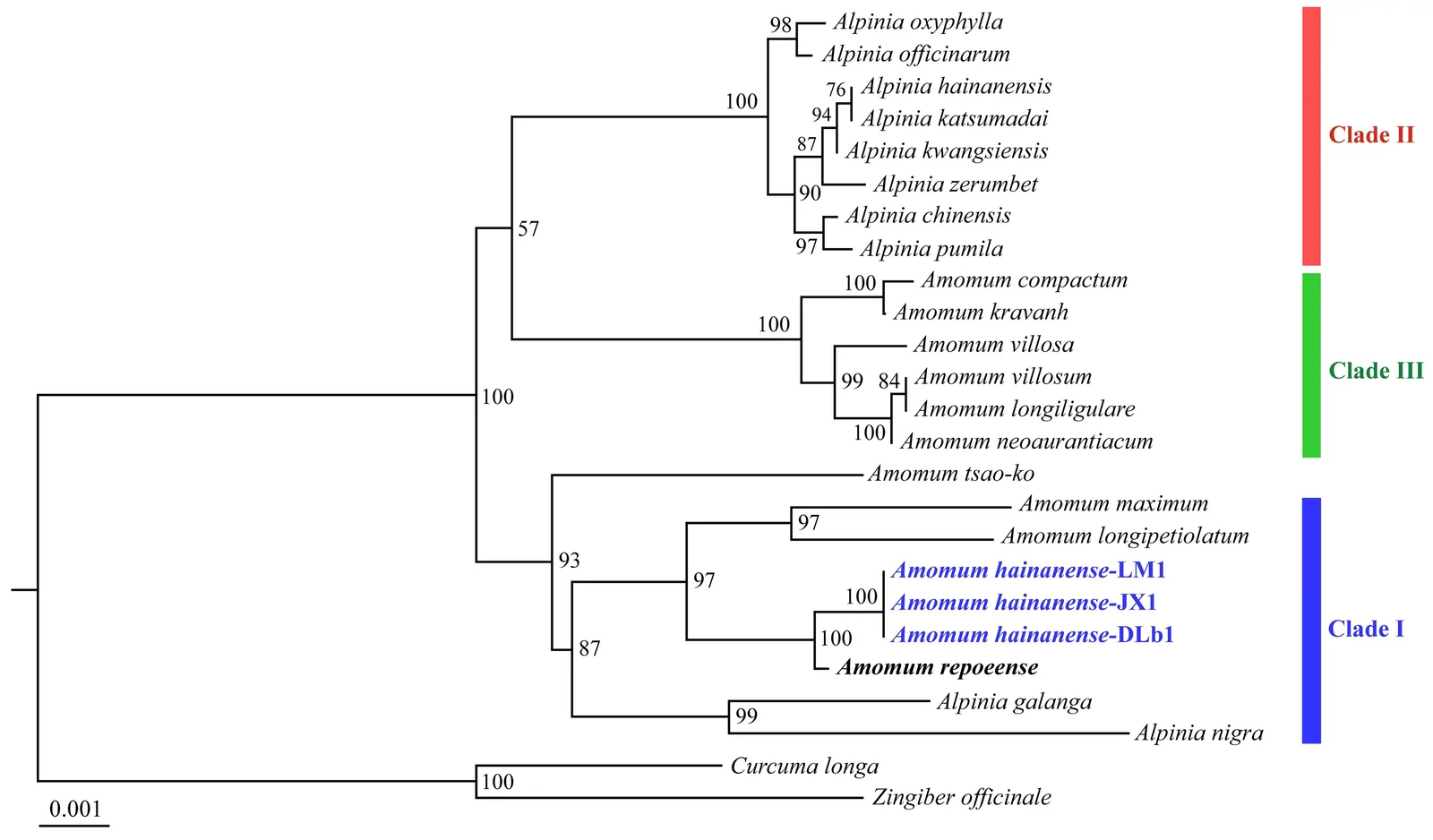
Maximum-likelihood (ML) phylogeny inferred from concatenated sequences of three plastid loci (*matK*, *rbcL* and *psbA-trnH*) including *A. repoeense* and taxa used in the plastome ML analyses. Numbers on branches are bootstrap support values. Accessions of *A. hainanense* are highlighted in blue, and coloured bars indicate Clades I–III. The scale bar indicates substitutions per site. *Zingiber officinale* and *Curcuma longa* were used as outgroups.

## Discussion

Understanding how genetic diversity is distributed within and among remnant populations is essential for conservation genomics of narrow-range island endemics, because restricted ranges, small population sizes and limited connectivity often lead to reduced genetic variation and increased genetic vulnerability (Frankham 1997, Caujape-Castells *et al.* 2010). In this study, we combined ddRAD-seq SNPs, complete plastome data and demographic modelling to assess the conservation genomic status and phylogenetic placement of *A. hainanense*. The results revealed extremely low nucleotide diversity, strong and significant population differentiation, and a long-term decline in effective population size. Together, these findings indicate severe genetic erosion and highlight the need for population-stratified conservation management.

### Phylogenetic placement of *Amomum hainanense*

Resolving the phylogenetic placement of an endemic species provides a necessary framework for discussing hypotheses about its origin. In the initial description of *Amomum hainanense*, Zou *et al.* (2018) proposed *A. repoeense* as its closest relative based on morphology, and this relationship is corroborated here by our phylogenetic analyses of three plastid loci (Fig. 5). Geographically, *A. repoeense* occurs from southern Yunnan into the Indochina Peninsula, whereas *A. hainanense* is restricted to montane forests on Hainan Island (Wu and Larsen 2000, Zou *et al.* 2018). Comparable allopatric sister relationships, in which a Hainan endemic species is recovered as sister to a geographically widespread lineage spanning Indochina and tropical southern China, have also been documented in other plant groups, including *Castanopsis* (Tang *et al.* 2025), *Machilus* (Huo *et al.* 2022) and *Mouretia* (Xu *et al.* 2026).

At broader floristic scales, Hainan exhibits strong biogeographic affinities with Indochina and tropical southern China. For instance, Zhu (2016) reported high floristic similarity between Hainan and Vietnam at both the family and genus levels. In a beta-diversity analysis of seed plant communities between Hainan Island and adjacent landmasses, Lin *et al.* (2021) revealed a hierarchical clustering pattern in which Hainan grouped first with Vietnam, then with Guangxi, Guangdong and Taiwan, and finally with the Philippines. Geological evidence also indicates long-term biogeographic connectivity among Hainan, Indochina and southern mainland China throughout much of the Cenozoic (Ali *et al.* 2025). However, the full opening of the Qiongzhou Strait during the early Holocene (ca. 11 ka BP) (Zhao *et al.* 2007, Jia *et al.* 2025) likely reinforced island–mainland isolation and reduced opportunities for cross-strait gene flow (Su *et al.* 2005). Nevertheless, the divergence time between *A. hainanense* and *A. repoeense* remains unresolved, precluding robust discrimination between two competing biogeographic hypotheses: vicariance driven by strait formation versus dispersal to Hainan followed by *in situ* isolation. Either process could generate the recovered sister relationship and the observed disjunct distributions between Hainan and the adjacent mainland. Future investigations incorporating expanded taxon sampling, time-calibrated phylogenies and explicit ancestral range reconstruction will be essential for testing these alternative hypotheses.

The plastome phylogeny also revealed a mixed placement of *Amomum* and *Alpinia* within Clade I, whereas the other two major clades were genus-restricted in our sampling (Fig. 4). This pattern should be interpreted cautiously because our taxon sampling was designed to determine the plastome placement of *A. hainanense* rather than to resolve generic boundaries within Alpinieae. Nevertheless, the mixed placement of *Amomum* and *Alpinia* is consistent with the long-recognized complexity of generic delimitation in Alpinieae, where previous molecular studies have shown *Alpinia* to be polyphyletic and *Amomum sensu* lato to require taxonomic recircumscription (Kress *et al.* 2005, 2007, de Boer *et al.* 2018). Such plastome patterns may reflect taxonomic complexity, plastid capture following historical hybridization, incomplete lineage sorting, or limited taxon sampling. Future phylogenomic studies using broader taxon sampling and independent nuclear genomic data will be needed to clarify whether plastome relationships are congruent with species-tree relationships.

### Low nucleotide diversity and high differentiation among populations

Assessing genetic diversity is fundamental to the conservation of endangered species because it underpins adaptive potential and informs management decisions (Frankham *et al.* 2010). However, genome-wide estimates of nucleotide diversity are available for only a few *Amomum* taxa. Amomi Fructus (‘Sharen’), a well-known traditional Chinese medicine, is derived from the dried mature fruits of *A. villosa* var. villosa (AVV), *A. villosa* var. *xanthioides* (AVX) and *A. longiligularis* (AL). Based on whole-genome resequencing, Chen *et al.* (2023) reported nucleotide diversity (*π*) values of 10.4 × 10^−4^ for AVX, 9.7 × 10^−4^ for AL, and 8.6 × 10^−4^ and 4.1 × 10^−4^ for the two geographic populations of AVV, AVV-JH and AVV-YC, respectively. By contrast, nucleotide diversity in the five well-sampled populations of *A. hainanense* was markedly lower, ranging from 0.7 × 10^−4^ to 1.7 × 10^−4^ across populations (Table 1), indicating severe genetic erosion in this endemic ginger. This pattern is consistent with genomic studies of endangered island endemic plants, in which small population size, restricted gene flow and historical bottlenecks have been linked to reduced genome-wide diversity and increased genetic vulnerability (Hamabata *et al.* 2019).

Chen *et al.* (2023) also estimated genetic differentiation among the three source taxa of Amomi Fructus. They reported high divergence between AL, which is endemic to Hainan Island, and taxa from Yunnan (AVX and AVV-JH) and Guangdong (AVV-YC) (*F*_ST_ = 0.579–0.684), intermediate divergence between AVX and AVV (*F*_ST_ = 0.273–0.394), and low differentiation between the two geographic populations of AVV (*F*_ST_ = 0.156). In our study, pairwise *F*_ST_ among the five well-sampled *A. hainanense* populations ranged from 0.280 to 0.621, with a mean of 0.457 (Table 2). Notably, even the lowest differentiation between *A. hainanense* populations (LM vs XL, *F*_ST_ = 0.280) was comparable to interspecific divergence reported for Amomi Fructus (AVX vs AVV-JH, *F*_ST_ = 0.273), highlighting pervasive population subdivision within *A. hainanense*.

The combination of exceptionally low within-population diversity and strong among-population differentiation is consistent with patterns frequently observed in threatened plants experiencing habitat fragmentation and long-term population size reduction (Aguilar *et al.* 2008, Schlaepfer *et al.* 2018). Stairway Plot 2 inferred a prolonged decline in effective population size (Fig. 3; see Supplementary Figure S2). Such long-term contraction is expected to intensify genetic drift, erode standing variation within populations and promote rapid divergence among isolated populations, yielding high *F*_ST_ even over modest geographic distances (Charlesworth 2009, Holsinger and Weir 2009, Frankham *et al.* 2010). As understorey herbs, *Amomum* species are commonly visited and pollinated by bees (Sharma *et al.* 2019, Yang *et al.* 2021). Because bee-mediated pollen dispersal is context-dependent, landscape fragmentation may limit pollinator movement such that effective pollen flow becomes increasingly localized within small, isolated patches (Hadley and Betts 2012, Aguilar *et al.* 2019). Reduced pollen-mediated gene flow would further strengthen population subdivision and increase inbreeding and drift, accelerating the loss of genetic variation (Ellstrand and Elam 1993, Young *et al.* 1996). Consistent with this expectation, positive inbreeding coefficients were detected in the well-sampled *A. hainanense* populations (Table 1). Overall, long-term demographic contraction and restricted connectivity among fragmented populations may have jointly driven the severe genetic erosion and pronounced population subdivision observed in *A. hainanense*. This finding is particularly important from a conservation perspective because it indicates that occurrence within protected forest landscapes does not necessarily prevent genetic erosion when remnant populations remain small and isolated. The conservation effectiveness for *A. hainanense* should therefore be evaluated not only by habitat protection, but also by whether practical conservation actions are implemented to maintain remaining genetic variation, assess the feasibility and risks of managed gene flow among isolated populations, and support population recovery (Frankham *et al.* 2010, Gramazio *et al.* 2020).

Nevertheless, the demographic inference should be interpreted with caution because the primary Stairway Plot 2 analysis was based on a folded SFS constructed from all sampled individuals. *A. hainanense* showed strong population subdivision (Fig. 2; Table 2), and pooling individuals from differentiated populations may distort the SFS and potentially generate signals that could be misinterpreted as changes in effective population size. Previous studies have shown that population structure and sampling scheme can strongly affect the SFS and that demographic models ignoring population subdivision may infer spurious population-size changes (Stadler *et al.* 2009, Chikhi *et al.* 2010, Mazet *et al.* 2016). To evaluate this potential bias, we repeated the Stairway Plot 2 analyses using folded SFSs constructed separately for two PCA-defined multi-population genetic groups. These group-based analyses recovered a broadly similar long-term decline in *N*_e_ [see Supplementary Figure S2], suggesting that the inferred declining trend is unlikely to be solely an artefact of pooling structured populations. Nevertheless, the exact timing and magnitude of the inferred *N*_e_ changes should be interpreted cautiously, and the Stairway Plot 2 results are best viewed as evidence for a broad historical decline rather than a precise reconstruction of population-size history.

## Implications for conservation

Our genome-wide SNP data provide an empirical basis for conserving the endangered Hainan endemic *A. hainanense*. The combination of extremely low nucleotide diversity, strong population differentiation and long-term demographic decline indicates that habitat protection alone is unlikely to be sufficient to maintain genetic variation in small, isolated populations; conservation should therefore incorporate explicit genetic monitoring, population-stratified germplasm collection and restoration management to improve conservation effectiveness (Frankham *et al.* 2010, Hamabata *et al.* 2019, Gramazio *et al.* 2020). Among the sampled populations, MLD and XL harboured the highest nucleotide diversity and heterozygosity and should therefore be prioritized for *in situ* conservation. We recommend defining conservation units (CUs) based primarily on the three PCA-defined genetic groups, which capture the dominant pattern of genetic differentiation and are broadly consistent with the neighbour-joining topology, rather than applying the highest supported STRUCTURE *K* directly to conservation-unit delimitation. For *ex situ* conservation, fruits or seeds should be collected in a population-stratified manner across all three PCA-defined genetic groups rather than only from the most diverse populations. Such collections could preserve both within-population diversity and among-population genetic differentiation, and provide genetic resources for propagation, research and future restoration. Given the strong inter-population differentiation, any translocation or assisted gene flow aimed at promoting outcrossing should preferably be restricted to populations within the same CU. Crosses between highly differentiated genetic groups should be considered only after controlled crossing experiments, common-garden evaluation or progeny fitness assessments indicate a low risk of outbreeding depression.

## Conclusions

Using genome-wide ddRAD-seq-derived SNPs and complete plastome assemblies, we provide a genomic assessment of the endangered island endemic *Amomum hainanense*. The species exhibits exceptionally low nucleotide diversity together with pronounced population structure and high among-population differentiation, indicating severe genetic erosion and restricted genetic connectivity among remnant populations. Demographic inference further suggests a long-term decline throughout the Holocene. Plastome phylogenomics supports *A. hainanense* as a well-supported monophyletic lineage, and three plastid loci identify *A. repoeense* as the closest sampled relative, providing a phylogenetic framework for testing alternative biogeographic hypotheses underlying its endemism. The evidence for marked genetic erosion in this nationally protected island endemic indicates that conservation planning should explicitly incorporate genetic diversity, population differentiation and the risks of inappropriate population mixing, rather than relying solely on the protected status of the species or habitat protection. Collectively, these results highlight the need for integrated *in situ* and *ex situ* conservation, including priority *in situ* protection of the most genetically diverse populations (MLD and XL) and the establishment of population-stratified germplasm collections to preserve remaining genetic variation and support future restoration.

## Supplementary Material

plag045_Supplementary_Data

## Data Availability

Raw sequencing reads generated in this study are publicly available in the NCBI Sequence Read Archive (SRA; BioProject accession PRJNA1442385; https://www.ncbi.nlm.nih.gov/sra/PRJNA1442385). Processed SNP datasets, files used for population structure and demographic analyses, complete chloroplast genome sequences, and final sequence alignments used for phylogenetic analyses are provided as individual Supporting Information files.
