# Supplementary material for: Genomic evidence for genetic erosion and strong population subdivision in Amomum hainanense (Zingiberaceae), an endangered island endemic plant from Hainan, China
Source: AoB Plants. 2026 Sep 24;18(5):plag045. doi: 10.1093/aobpla/plag045 (PMC13643486; doi:10.1093/aobpla/plag045)
Supplement: Supplementary file 1 [file plag045_supplementary_data.zip › Supporting Information.pdf]

Table S1. Effects of different values of the *M* parameter in *ustacks* on RAD locus assembly and SNP retention in the Stacks pipeline.

M	1	2	3	4	5	6
Retained RAD loci	36882	32624	29961	28125	26646	25392
Polymorphic RAD loci	8716	8271	7796	7337	6916	6515
SNPs	12702	13372	13741	13791	13907	14059
0 SNP loci	28166	24353	22165	20788	19730	18877
1 SNP loci	6322	5397	4919	4528	4179	3897
2 SNP loci	1576	1766	1568	1434	1337	1207
3 SNP loci	496	641	663	627	588	554
4–5 SNP loci	228	342	430	463	434	415
6–10 SNP loci	79	100	175	222	285	320
>10 SNP loci	15	25	41	63	93	122

For each *M* value, the catalog-building parameter *n* was set to the same value as *M*. The row “SNPs” refers to filtered SNPs before applying the one-random-SNP-per-locus option. The final one-random-SNP dataset for $M = 1$ contained 8,716 SNPs.

Table S2. Number of clean read pairs retained for each accession of *Amomum hainanense*.

Populations	Samples	Numbers of clean read pairs generated by ddRAD-seq
NK	NK1	3111196
YG	YG1	2717067
DL	Dla2	5007750
	Dla3	2608933
	Dla4	3133158
	Dlb1	3153862
	Dlb2	3274487
	Dlb3	3222754
	Dlb4	3149558
	Dlb5	3119017
	Dlb6	3069688
	Dlb7	7576004
	Dlc1	2826005
	Dlc2	7061916
	Dlc3	2294151
	Dlc4	2802579

	Dlc5	1635848
JX	JX1	2599404
	JX2	2849895
	JX3	1962474
LM	LM1	2693256
	LM2	3014515
	LM3	2788975
	LM4	2737767
	LM5	2669343
	LM6	2699243
	LM7	3221482
	LM8	2758180
	LM9	2913179
	LM10	3018302
	LM11	2874653
	LM12	2759558
	LM13	2911784
	LM14	2715153
	LM15	2765618
MLD	MLD1	2569649
	MLD2	2004965
	MLD3	2952844
	MLD4	3030484
	MLD5	1369213
	MLD6	922533
	MLD7	2943906
WZ	WZ1	3204586
	WZ2	3328072
	WZ3	1777170
	WZ4	3122050
	WZ5	3291316
	WZ6	1175014
	WZ7	2694081
	WZ8	3185263
	WZ9	3392651
	WZ10	3327083
	WZ11	3418980
	WZ12	3094369
	WZ13	3312068
	WZ14	3245392
	WZ15	2495356
XL	Xla1	2930041
	Xla2	3009093
	Xla3	3198242

Xla4	2853001
Xla5	3205074
Xla6	2746247
Xlb1	2749279
Xlb2	2858693
Xlb3	2833812
Xlb4	2905345
Xlb5	2037486
Xlb6	1683767

Figure S1. Effective population size (N_e) trajectories inferred with Stairway Plot 2 using alternative generation times (T) and two SFS construction approaches. Solid lines represent the median estimates of N_e and shaded areas indicate 95% confidence intervals.

(a) easySFS, $T = 2$ years; (b) easySFS, $T = 4$ years; (c) vcf2sfs, $T = 2$ years; (d) vcf2sfs, $T = 4$ years.

Alt text: Four Stairway Plot 2 panels showing inferred effective population size trajectories of *Amomum hainanense* under alternative generation times and two SFS construction approaches.

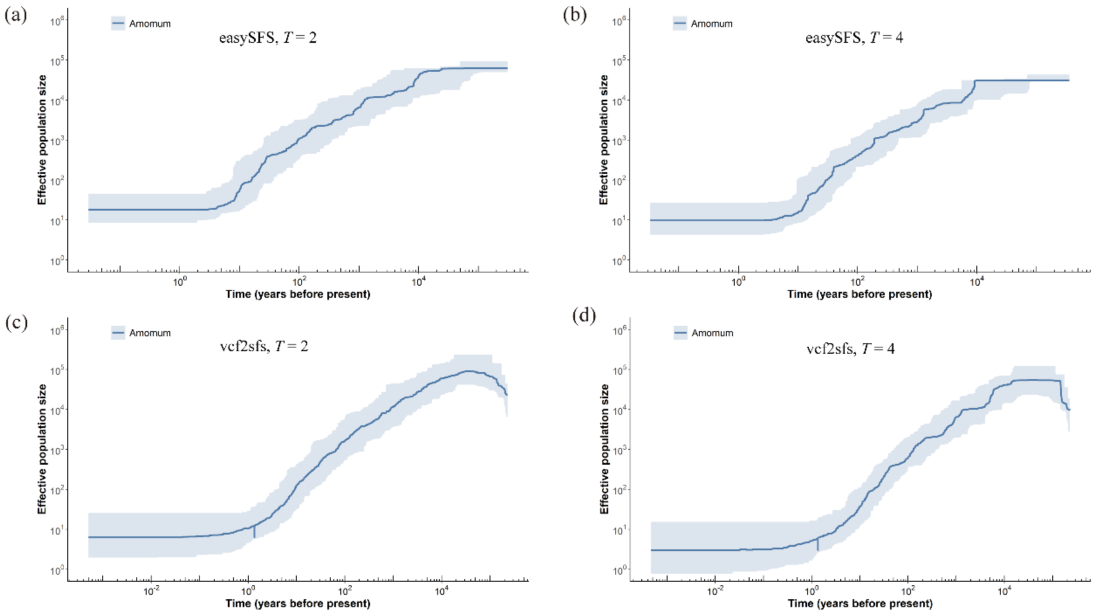

Figure S2. Effective population size (N_e) trajectories inferred with Stairway Plot 2 based on two PCA-defined multi-population genetic groups (generation time T = 3 years). Solid lines represent median estimates of N_e and shaded areas indicate 95% confidence intervals.

(a) easySFS, group II (LM and XL); (b) vcf2sfs, group II (LM and XL); (c) easySFS, a subset of group III (DL, WZ and JX); (d) vcf2sfs, a subset of group III (DL, WZ and JX).

Alt text: Four Stairway Plot 2 panels showing effective population size trajectories inferred separately for two PCA-defined multi-population genetic groups of *Amomum hainanense*.

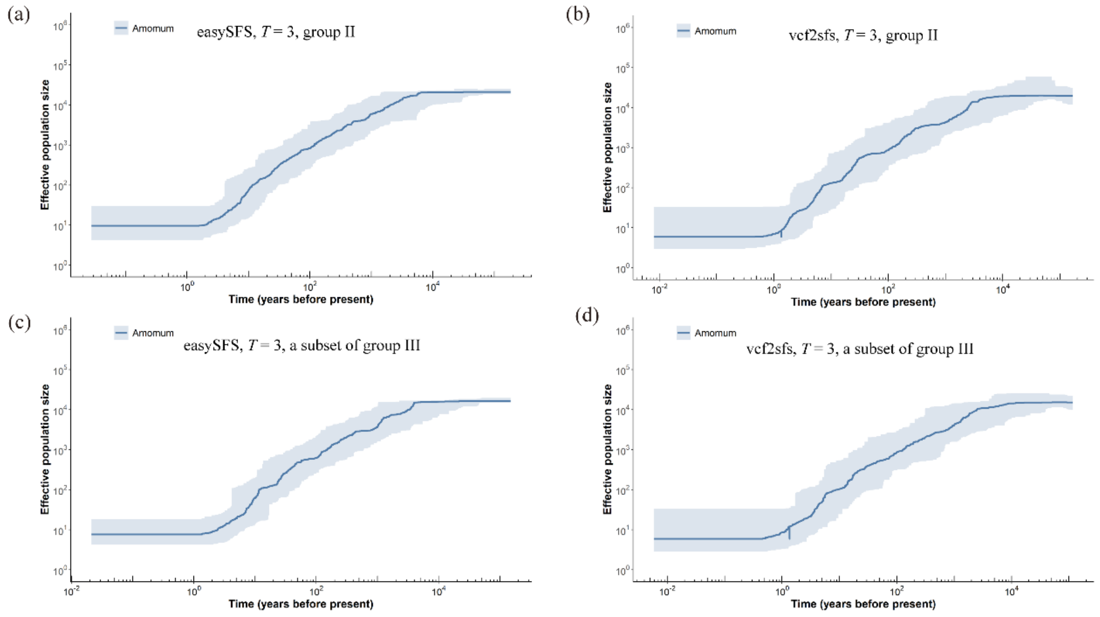

List of Individual Supporting Information files

all.snp.vcf: VCF dataset containing all SNPs retained after filtering.

random.snp.vcf: A linkage-reduced SNP dataset generated using the *--write-random-snp* option in the Stacks *populations* program to retain one randomly selected SNP per RAD locus.

STRUCTURE_input: Genotype input file used for the STRUCTURE analysis.

A_hainanense_22_plastomes.fasta: Final alignment of complete chloroplast genome sequences from 22 accessions of *Amomum hainanense*, used for estimating intraspecific chloroplast nucleotide diversity.

Amomum_Fig4_alignment.fasta: Final complete-plastome alignment used for the phylogenetic analysis shown in Figure 4.

Amomum_Fig5_alignment.fasta: Final concatenated alignment of three plastid loci (*matK*, *rbcL* and *psbA-trnH*) used for the phylogenetic analysis shown in Figure 5.

Population assignment table linking each individual to its sampling population.

Samples	Populations
Dla2	DL
Dla3	DL
Dla4	DL
Dlb1	DL
Dlb2	DL
Dlb3	DL
Dlb4	DL
Dlb5	DL
Dlb6	DL
Dlb7	DL
Dlc1	DL
Dlc2	DL
Dlc3	DL
Dlc4	DL
Dlc5	DL
JX1	JX
JX2	JX
JX3	JX
LM1	LM
LM2	LM
LM3	LM
LM4	LM
LM5	LM
LM6	LM
LM7	LM
LM8	LM
LM9	LM
LM10	LM

LM11	LM
LM12	LM
LM13	LM
LM14	LM
LM15	LM
MLD1	ML
MLD2	ML
MLD3	ML
MLD4	ML
MLD5	ML
MLD6	ML
MLD7	ML
NK1	NK
WZ1	WZ
WZ2	WZ
WZ3	WZ
WZ4	WZ
WZ5	WZ
WZ6	WZ
WZ7	WZ
WZ8	WZ
WZ9	WZ
WZ10	WZ
WZ11	WZ
WZ12	WZ
WZ13	WZ
WZ14	WZ
WZ15	WZ
Xla1	XL
Xla2	XL
Xla3	XL
Xla4	XL
Xla5	XL
Xla6	XL
Xlb1	XL
Xlb2	XL
Xlb3	XL
Xlb4	XL
Xlb5	XL
Xlb6	XL
YG1	YG

STRUCTURE parameter settings

Parameter settings used for the STRUCTURE analysis, including the contents of the mainparams and extraparams files.

mainparams file:

```
KEY PARAMETERS FOR THE PROGRAM structure.  YOU WILL NEED TO SET THESE
IN ORDER TO RUN THE PROGRAM.  VARIOUS OPTIONS CAN BE ADJUSTED IN THE
FILE extraparams.

(int) means that this takes an integer value.
(B)   means that this variable is Boolean
       (ie insert 1 for True, and 0 for False)
(str) means that this is a string (but not enclosed in quotes!)

Basic Program Parameters
#define MAXPOPS   12       // (int) number of populations assumed
#define BURNIN    100000   // (int) length of burnin period
#define NUMREPS   200000   // (int) number of MCMC reps after burnin

Input/Output files
#define INFILE   infile    // (str) name of input data file
#define OUTFILE  outfile   // (str) name of output data file

Data file format
#define NUMINDS   69      // (int) number of diploid individuals in data file
#define NUMLOCI   8688    // (int) number of loci in data file
#define PLOIDY    2       // (int) ploidy of data
#define MISSING   0       // (int) value given to missing genotype data
#define ONEROWPERIND 0    // (B) store data for individuals in a single line


#define LABEL     1     // (B) Input file contains individual labels
#define POPDATA   1     // (B) Input file contains a population identifier
#define POPFLAG   0     // (B) Input file contains a flag which says
                            whether to use popinfo when USEPOPINFO==1
#define LOCDATA   0     // (B) Input file contains a location identifier

#define PHENOTYPE 0     // (B) Input file contains phenotype information
#define EXTRACOLS 0     // (int) Number of additional columns of data
                            before the genotype data start.

#define MARKERNAMES      0  // (B) data file contains row of marker names
```

```
#define RECESSIVEALLELES 0  // (B) data file contains dominant markers (eg AFLPs)
                            // and a row to indicate which alleles are recessive
#define MAPDISTANCES     0  // (B) data file contains row of map distances
                           // between loci
```

Advanced data file options

```
#define PHASED            0 // (B) Data are in correct phase (relevant for linkage model only)
#define PHASEINFO         0 // (B) the data for each individual contains a line
                                 indicating phase (linkage model)
#define MARKOVPHASE       0 // (B) the phase info follows a Markov model.
#define NOTAMBIGUOUS   -999 // (int) for use in some analyses of polyploid data
```

extraparams file:

EXTRA PARAMS FOR THE PROGRAM structure. THESE PARAMETERS CONTROL HOW THE PROGRAM RUNS. ATTRIBUTES OF THE DATAFILE AS WELL AS K AND RUNLENGTH ARE SPECIFIED IN mainparams.

(int) means that this takes an integer value.

"(d)   means that this is a double (ie, a Real number such as 3.14)."

(B)   means that this variable is Boolean

"   (ie insert 1 for True, and 0 for False)."

PROGRAM OPTIONS

```
#define NOADMIX    0 // (B) Use no admixture model (0=admixture model, 1=no-admix)
#define LINKAGE    0 // (B) Use the linkage model model
#define USEPOPINFO  0 // (B) Use prior population information to pre-assign
                              individuals to clusters
#define LOCPRIOR   0 //(B)  Use location information to improve weak data
#define FREQSCORR   1 // (B) allele frequencies are correlated among pops
#define ONEFST     0 // (B) assume same value of Fst for all subpopulations.
#define INFERALPHA  1 // (B) Infer ALPHA (the admixture parameter)
#define POPALPHAS   0 // (B) Individual alpha for each population
#define ALPHA     1.0 // (d) Dirichlet parameter for degree of admixture
                           (this is the initial value if INFERALPHA==1).
#define INFERLAMBDA 0 // (B) Infer LAMBDA (the allele frequencies parameter)
#define POPSPECIFICLAMBDA 0 //(B) infer a separate lambda for each pop
                              (only if INFERLAMBDA=1).
#define LAMBDA    1.0 // (d) Dirichlet parameter for allele frequencies
```

PRIORS

```
#define FPRIORMEAN 0.01  // (d) Prior mean and SD of Fst for pops.
#define FPRIORSD   0.05  // (d) The prior is a Gamma distribution with these parameters
#define UNIFPRIORALPHA 1 // (B) use a uniform prior for alpha;
                                otherwise gamma prior
#define ALPHAMAX     10.0 // (d) max value of alpha if uniform prior
#define ALPHAPRIORA   1.0 // (only if UNIFPRIORALPHA==0): alpha has a gamma
"                            prior with mean A*B, and "
#define ALPHAPRIORB   2.0 // variance A*B^2.

#define LOG10RMIN     -4.0   //(d) Log10 of minimum allowed value of r under linkage
                                   model
#define LOG10RMAX      1.0   //(d) Log10 of maximum allowed value of r
#define LOG10RPROPSD   0.1   //(d) standard deviation of log r in update
#define LOG10RSTART   -2.0   //(d) initial value of log10 r
```

USING PRIOR POPULATION INFO (USEPOPINFO)

```
#define GENSBACK    2  //(int) For use when inferring whether an indiv-
"                       idual is an immigrant, or has an immigrant an-"
"                       cestor in the past GENSBACK generations.  eg, if "
"                       GENSBACK==2, it tests for immigrant ancestry "
                        back to grandparents.
#define MIGRPRIOR 0.01 //(d) prior prob that an individual is a migrant
                          (used only when USEPOPINFO==1).  This should
"                         be small, eg 0.01 or 0.1."
#define PFROMPOPFLAGONLY 0 // (B) only use individuals with POPFLAG=1 to update  P.
                              This is to enable use of a reference set of
"                             individuals for clustering additional ""test"" "
                              individuals.
```

LOCPRIOR MODEL FOR USING LOCATION INFORMATION

```
#define LOCISPOP      0    //(B) use POPDATA for location information
"#define LOCPRIORINIT  1.0  //(d) initial value for r, the location prior"
#define MAXLOCPRIOR  20.0  //(d) max allowed value for r
```

OUTPUT OPTIONS

```
"#define PRINTNET     1 // (B) Print the ""net nucleotide distance"" to screen during
the run"
#define PRINTLAMBDA  1 // (B) Print current value(s) of lambda to screen
#define PRINTQSUM    1 // (B) Print summary of current population membership to screen
#define SITEBYSITE   0  // (B) whether or not to print site by site results.
                        (Linkage model only) This is a large file!
#define PRINTQHAT    0  // (B) Q-hat printed to a separate file.  Turn this
```

```
                        on before using STRAT.
#define UPDATEFREQ   100  // (int) frequency of printing update on the screen.
                             Set automatically if this is 0.
#define PRINTLIKES   1  // (B) print current likelihood to screen every rep
#define INTERMEDSAVE 1  // (int) number of saves to file during run
#define ECHODATA     1  // (B) Print some of data file to screen to check
                           that the data entry is correct.

(NEXT 3 ARE FOR COLLECTING DISTRIBUTION OF Q:)
#define ANCESTDIST   0  // (B) collect data about the distribution of an-
                           cestry coefficients (Q) for each individual
#define NUMBOXES   1000 // (int) the distribution of Q values is stored as
                           a histogram with this number of boxes.
#define ANCESTPINT 0.90 // (d) the size of the displayed probability
                           interval on Q (values between 0.0--1.0)

MISCELLANEOUS
#define COMPUTEPROB 1     // (B) Estimate the probability of the Data under
                          the model.  This is used when choosing the
                          best number of subpopulations.
#define ADMBURNIN  500    // (int) [only relevant for linkage model]:
                          Initial period of burnin with admixture model (see Readme)
#define ALPHAPROPSD 0.025 // (d) SD of proposal for updating alpha
#define STARTATPOPINFO 0  // Use given populations as the initial condition
                          for population origins.  (Need POPDATA==1).  It
                          is assumed that the PopData in the input file
                          are between 1 and k where k<=MAXPOPS.
#define RANDOMIZE      0  // (B) use new random seed for each run
#define SEED       2245  // (int) seed value for random number generator
                       (must set RANDOMIZE=0)
#define METROFREQ    10   // (int) Frequency of using Metropolis step to update
                          Q under admixture model (ie use the metr. move every
"                          i steps).  If this is set to 0, it is never used."
                          (Proposal for each q^(i) sampled from prior.  The
                          goal is to improve mixing for small alpha.)
#define REPORTHITRATE 0 //   (B) report hit rate if using METROFREQ
```

The command lines used to generate SFS by easySFS

(1) random.snp.vcf

```
/home/tangl/share/0soft/easySFS/easySFS.py -i random.snp.vcf -p pop.map --preview
```

```
-------------------------------------------------------------------------- screen out
Processing 1 populations - odict_keys(['Amomum'])
  Sampling one snp per locus

    Running preview mode. We will print out the results for # of segregating sites
    for multiple values of projecting down for each population. The dadi
    manual recommends maximizing the # of seg sites for projections, but also
    a balance must be struck between # of seg sites and sample size.

    For each population you should choose the value of the projection that looks
    best and then rerun easySFS with the `--proj` flag.

Amomum
(2, 2923)  (3, 4385)  (4, 5277)  (5, 5883)  (6, 6325)  (7, 6663)  (8, 6930)  (9, 7147)  (10, 7327)
(11, 7478)  (12, 7607)  (13, 7717)  (14, 7814)  (15, 7898)  (16, 7972)  (17, 8037)  (18, 8095)  (19, 8147)
(20, 8193)  (21, 8235)  (22, 8272)  (23, 8305)  (24, 8335)  (25, 8361)  (26, 8386)  (27, 8409)  (28, 8429)
(29, 8448)  (30, 8465)  (31, 8480)  (32, 8495)  (33, 8508)  (34, 8520)  (35, 8528)  (36, 8538)  (37, 8546)
(38, 8555)  (39, 8560)  (40, 8567)  (41, 8572)  (42, 8578)  (43, 8563)  (44, 8569)  (45, 8547)  (46, 8552)
(47, 8512)  (48, 8516)  (49, 8476)  (50, 8479)  (51, 8464)  (52, 8467)  (53, 8461)  (54, 8463)  (55, 8427)
(56, 8429)  (57, 8372)  (58, 8374)  (59, 8294)  (60, 8296)  (61, 8224)  (62, 8225)  (63, 8149)  (64, 8150)
(65, 8074)  (66, 8075)  (67, 8015)  (68, 8016)  (69, 7935)  (70, 7936)  (71, 7852)  (72, 7853)  (73, 7772)
(74, 7772)  (75, 7678)  (76, 7678)  (77, 7588)  (78, 7589)  (79, 7515)  (80, 7516)  (81, 7408)  (82, 7408)
(83, 7300)  (84, 7300)  (85, 7188)  (86, 7188)  (87, 7088)  (88, 7089)  (89, 6991)  (90, 6991)  (91, 6878)
(92, 6878)  (93, 6760)  (94, 6760)  (95, 6665)  (96, 6665)  (97, 6567)  (98, 6567)  (99, 6465)  (100, 6465)
(101, 6311) (102, 6311) (103, 6176) (104, 6176) (105, 6036) (106, 6036) (107, 5880) (108, 5880) (109, 5770)
(110, 5770) (111, 5673) (112, 5673) (113, 5568) (114, 5568) (115, 5439) (116, 5439) (117, 5286) (118, 5286)
(119, 5108) (120, 5108) (121, 4927) (122, 4927) (123, 4722) (124, 4722) (125, 4472) (126, 4472) (127, 4247)
(128, 4247) (129, 3975) (130, 3975) (131, 3606) (132, 3606) (133, 3147) (134, 3147) (135, 2491) (136, 2491)
(137, 1562) (138, 1562)

/home/tangl/share/0soft/easySFS/easySFS.py -i random.snp.vcf -p pop.map --proj 42
--------------------------------------------------------------------------screen out
Processing 1 populations - odict_keys(['Amomum'])
  Sampling one snp per locus
Doing 1D sfs - Amomum
Doing multiSFS for all pops
```

(2) groupII.snps.vcf, the vcf file generated using individuals from group II identified

by PCA.

```
/home/tangl/share/0soft/easySFS/easySFS.py -i groupII.snps.vcf -p pop-II.map --preview
```

```
------------------------------------------------------------------------- screen out
Processing 1 populations - odict_keys(['Amomum'])
  Sampling one snp per locus

    Running preview mode. We will print out the results for # of segregating sites
    for multiple values of projecting down for each population. The dadi
    manual recommends maximizing the # of seg sites for projections, but also
    a balance must be struck between # of seg sites and sample size.

    For each population you should choose the value of the projection that looks
    best and then rerun easySFS with the `--proj` flag.

Amomum
(2, 1615) (3, 2422) (4, 2923) (5, 3271) (6, 3530) (7, 3734) (8, 3899) (9, 4036) (10, 4153)(11, 4254)
(12, 4341)(13, 4417)(14, 4485)(15, 4544)(16, 4597)(17, 4644)(18, 4686)(19, 4723)(20, 4756)(21, 4785)
(22, 4811)(23, 4835)(24, 4855)(25, 4873)(26, 4889)(27, 4904)(28, 4916)(29, 4927)(30, 4936)(31, 4944)
(32, 4951)(33, 4957)(34, 4963)(35, 4967)(36, 4970)(37, 4973)(38, 4976)(39, 4978)(40, 4980)(41, 4981)
(42, 4982)(43, 4982)(44, 4983)(45, 4745)(46, 4746)(47, 4295)(48, 4295)(49, 3740)(50, 3740)(51, 3031)
(52, 3031)(53, 2052)(54, 2052)
```

```
/home/tangl/share/0soft/easySFS/easySFS.py -i groupII.snps.vcf -p pop-II.map --proj 44
```

```
------------------------------------------------------------------------- screen out
Processing 1 populations - odict_keys(['Amomum'])
  Sampling one snp per locus
Doing 1D sfs - Amomum
Doing multiSFS for all pops
```

(2) groupIII.snps.vcf, the vcf file generated using individuals from group III identified by PCA.

```
/home/tangl/share/0soft/easySFS/easySFS.py -i groupIII.snps.vcf -p pop-III.map --preview
```

```
------------------------------------------------------------------------- screen out
Processing 1 populations - odict_keys(['Amomum'])
  Sampling one snp per locus
```

```
Running preview mode. We will print out the results for # of segregating sites
for multiple values of projecting down for each population. The dadi
manual recommends maximizing the # of seg sites for projections, but also
a balance must be struck between # of seg sites and sample size.

For each population you should choose the value of the projection that looks
best and then rerun easySFS with the `--proj` flag.
```

```
Amomum
(2, 909)  (3, 1364) (4, 1644) (5, 1837) (6, 1979) (7, 2089) (8, 2178) (9, 2251) (10, 2313)
(11, 2366)(12, 2412)(13, 2452)(14, 2487)(15, 2517)(16, 2545)(17, 2569)(18, 2591)(19, 2610)
(20, 2628)(21, 2643)(22, 2657)(23, 2670)(24, 2681)(25, 2691)(26, 2700)(27, 2708)(28, 2716)
(29, 2722)(30, 2728)(31, 2733)(32, 2738)(33, 2742)(34, 2746)(35, 2749)(36, 2752)(37, 2754)
(38, 2756)(39, 2758)(40, 2760)(41, 2761)(42, 2763)(43, 2764)(44, 2764)(45, 2765)(46, 2766)
(47, 2766)(48, 2767)(49, 2767)(50, 2767)(51, 2768)(52, 2768)(53, 2768)(54, 2768)(55, 2719)
(56, 2719)(57, 2604)(58, 2604)(59, 2399)(60, 2399)(61, 2113)(62, 2113)(63, 1775)(64, 1775)
(65, 1191)(66, 1191)
```

```
/home/tangl/share/0soft/easySFS/easySFS.py -i groupIII.snps.vcf -p pop-III.map --proj
54
------------------------------------------------------------------------- screen out
Processing 1 populations - odict_keys(['Amomum'])
  Sampling one snp per locus
Doing 1D sfs - Amomum
Doing multiSFS for all pops
```

The command lines used to generate SFS by vcf2sfs

(1) random.snp.vcf

```
Amgt<-vcf2gt("random.snp.vcf", "pop.map")
Amsfs1<-gt2sfs.raw(Amgt, "Amomum")
write.table(Am69sfs1, file = "Amsfs1.txt", sep = "\t", row.names = FALSE, col.names
= TRUE, quote = FALSE, fileEncoding = "UTF-8")
```

(2) groupII.snps.vcf, the vcf file generated using individuals from group II identified by PCA.

```
AmIIgt<-vcf2gt("groupII.snps.vcf", "pop-II.map")
```

```
AmIIsfs1<-gt2sfs.raw(AmIIgt, "Amomum")

write.table(AmIIsfs1, file = "AmIIsfs1.txt", sep = "\t", row.names = FALSE, col.names = TRUE, quote = FALSE, fileEncoding = "UTF-8")
```

(3) groupIII.snps.vcf, the vcf file generated using individuals from group III identified by PCA.

```
AmIIIgt<-vcf2gt("groupIII.snps.vcf", "pop-III.map")

AmIIIsfs1<-gt2sfs.raw(AmIIIgt, "Amomum")

write.table(AmIIIsfs1, file = "AmIIIsfs1.txt", sep = "\t", row.names = FALSE, col.names = TRUE, quote = FALSE, fileEncoding = "UTF-8")
```

The blueprint files used for Stairway Plot 2 analyses to produce Figure 3.

The blueprint file using SFS generated by easySFS, assuming a generation time of 3 years:

```
#example blueprint file

#input setting

popid: Amomum # id of the population (no white space)

nseq: 42 # number of sequences

L: 12236258 # total number of observed nucleic sites, including polymorphic and monomorphic

whether_folded: true # whethr the SFS is folded (true or false)

SFS: 258.136742109628 449.2940687916999 566.2317347864682 571.1796932172283 523.7254685089665 480.758916090245 446.1899858962258 427.3384310801068 408.3360926478773 393.8931447594986 381.4323325075878 374.8525032716202 371.6810228047174 376.9213533358181 383.1907716719851 392.4993882376692 396.3692378400299 396.8572097027486 392.8158041431753 391.5095331747196 194.5921657556521 # snp frequency spectrum: number of singleton, number of doubleton, etc. (separated by white space)

#smallest_size_of_SFS_bin_used_for_estimation: 1 # default is 1; to ignore singletons, uncomment this line and change this number to 2

#largest_size_of_SFS_bin_used_for_estimation: 21 # default is nseq/2 for folded SFS

pct_training: 0.67 # percentage of sites for training

nrand: 10 20 30 40 # number of random break points for each try (separated by white space)

project_dir: Amomum_fold # project directory

stairway_plot_dir: stairway_plot_es # directory to the stairway plot files

ninput: 200 # number of input files to be created for each estimation

#random_seed: 6

#output setting

mu: 2.2167e-9 # assumed mutation rate per site per generation
```

```
year_per_generation: 3 # assumed generation time (in years). The mu = 7.389e-10 * 3 = 2.2167e-9

#plot setting

plot_title: Amomum_fold # title of the plot

xrange: 0,0 # Time (1k year) range; format: xmin,xmax; "0,0" for default

yrange: 0,0 # Ne (1k individual) range; format: xmin,xmax; "0,0" for default

xspacing: 5 # X axis spacing

yspacing: 5 # Y axis spacing

fontsize: 12 # Font size
```

The blueprint file using SFS generated by vcf2sfs, assuming a generation time of 3 years:

```
#example blueprint file

#input setting

popid: Amomum # id of the population (no white space)

nseq: 138 # number of sequences

L: 12236258 # total number of observed nucleic sites, including polymorphic and monomorphic

whether_folded: true # whethr the SFS is folded (true or false)

SFS: 52 71 85 181 96 293 121 319 101 295 99 377 69 243 71 297 98 324 87 173 89 232 83 300 83 248 69 251 77 225 70 268 50 140 51 121 46 126 35 155 65 169 51 114 48 128 55 188 55 124 57 183 81 289 50 97 24 75 28 133 46 98 25 101 15 102 13 74 6 # snp frequency spectrum: number of singleton, number of doubleton, etc. (separated by white space)

#smallest_size_of_SFS_bin_used_for_estimation: 1 # default is 1; to ignore singletons, uncomment this line and change this number to 2

#largest_size_of_SFS_bin_used_for_estimation: 69 # default is nseq/2 for folded SFS

pct_training: 0.67 # percentage of sites for training

nrand: 34 68 102 136 # number of random break points for each try (separated by white space)

project_dir: Amomum_fold # project directory

stairway_plot_dir: stairway_plot_es # directory to the stairway plot files

ninput: 200 # number of input files to be created for each estimation

#random_seed: 6

#output setting

mu: 2.2167e-9 # assumed mutation rate per site per generation

year_per_generation: 3 # assumed generation time (in years). The mu = 7.389e-10 * 3 = 2.2167e-9

#plot setting

plot_title: Amomum_fold # title of the plot
```

```
xrange: 0,0 # Time (1k year) range; format: xmin,xmax; "0,0" for default
yrange: 0,0 # Ne (1k individual) range; format: xmin,xmax; "0,0" for default
xspacing: 5 # X axis spacing
yspacing: 5 # Y axis spacing
fontsize: 12 # Font size
```

The blueprint files used for Stairway Plot 2 analyses to produce Figure S1.

The blueprint file using SFS generated by easySFS, assuming a generation time of 2 years:

```
#example blueprint file
#input setting
popid: Amomum # id of the population (no white space)
nseq: 42 # number of sequences
L: 12236258 # total number of observed nucleic sites, including polymorphic and monomorphic
whether_folded: true # whethr the SFS is folded (true or false)
SFS: 258.136742109628 449.2940687916999 566.2317347864682 571.1796932172283 523.7254685089665 480.758916090245 446.1899858962258 427.3384310801068 408.3360926478773 393.8931447594986 381.4323325075878 374.8525032716202 371.6810228047174 376.9213533358181 383.1907716719851 392.4993882376692 396.3692378400299 396.8572097027486 392.8158041431753 391.5095331747196 194.5921657556521 # snp frequency spectrum: number of singleton, number of doubleton, etc. (separated by white space)
#smallest_size_of_SFS_bin_used_for_estimation: 1 # default is 1; to ignore singletons, uncomment this line and change this number to 2
#largest_size_of_SFS_bin_used_for_estimation: 21 # default is nseq/2 for folded SFS
pct_training: 0.67 # percentage of sites for training
nrand: 10 20 30 40 # number of random break points for each try (separated by white space)
project_dir: Amomum_fold # project directory
stairway_plot_dir: stairway_plot_es # directory to the stairway plot files
ninput: 200 # number of input files to be created for each estimation
#random_seed: 6
#output setting
mu: 1.4778e-9 # assumed mutation rate per site per generation
year_per_generation: 2 # assumed generation time (in years). The mu = 7.389e-10 * 2 = 1.4778e-9
#plot setting
plot_title: Amomum_fold # title of the plot
xrange: 0,0 # Time (1k year) range; format: xmin,xmax; "0,0" for default
```

```
yrange: 0,0 # Ne (1k individual) range; format: xmin,xmax; "0,0" for default
xspacing: 5 # X axis spacing
yspacing: 5 # Y axis spacing
fontsize: 12 # Font size
```

The blueprint file using SFS generated by easySFS, assuming a generation time of 4 years:

```
#example blueprint file
#input setting
popid: Amomum # id of the population (no white space)
nseq: 42 # number of sequences
L: 12236258 # total number of observed nucleic sites, including polymorphic and monomorphic
whether_folded: true # whethr the SFS is folded (true or false)
SFS: 258.136742109628 449.2940687916999 566.2317347864682 571.1796932172283 523.7254685089665 480.758916090245 446.1899858962258 427.3384310801068 408.3360926478773 393.8931447594986 381.4323325075878 374.8525032716202 371.6810228047174 376.9213533358181 383.1907716719851 392.4993882376692 396.3692378400299 396.8572097027486 392.8158041431753 391.5095331747196 194.5921657556521 # snp frequency spectrum: number of singleton, number of doubleton, etc. (separated by white space)
#smallest_size_of_SFS_bin_used_for_estimation: 1 # default is 1; to ignore singletons, uncomment this line and change this number to 2
#largest_size_of_SFS_bin_used_for_estimation: 21 # default is nseq/2 for folded SFS
pct_training: 0.67 # percentage of sites for training
nrand: 10 20 30 40 # number of random break points for each try (separated by white space)
project_dir: Amomum_fold # project directory
stairway_plot_dir: stairway_plot_es # directory to the stairway plot files
ninput: 200 # number of input files to be created for each estimation
#random_seed: 6
#output setting
mu: 2.9556e-9 # assumed mutation rate per site per generation
year_per_generation: 4 # assumed generation time (in years). The mu = 7.389e-10 * 4 = 2.9556e-9
#plot setting
plot_title: Amomum_fold # title of the plot
xrange: 0,0 # Time (1k year) range; format: xmin,xmax; "0,0" for default
yrange: 0,0 # Ne (1k individual) range; format: xmin,xmax; "0,0" for default
xspacing: 5 # X axis spacing
```

```
yspacing: 5 # Y axis spacing
fontsize: 12 # Font size
```

The blueprint file using SFS generated by vcf2sfs, assuming a generation time of 2 years:

```
#example blueprint file
#input setting
popid: Amomum # id of the population (no white space)
nseq: 138 # number of sequences
L: 12236258 # total number of observed nucleic sites, including polymorphic and monomorphic
whether_folded: true # whethr the SFS is folded (true or false)
SFS: 52 71 85 181 96 293 121 319 101 295 99 377 69 243 71 297 98 324 87 173 89 232 83 300 83 248 69 251 77 225 70 268 50 140 51 121 46 126 35 155 65 169 51 114 48 128 55 188 55 124 57 183 81 289 50 97 24 75 28 133 46 98 25 101 15 102 13 74 6 # snp frequency spectrum: number of singleton, number of doubleton, etc. (separated by white space)
#smallest_size_of_SFS_bin_used_for_estimation: 1 # default is 1; to ignore singletons, uncomment this line and change this number to 2
#largest_size_of_SFS_bin_used_for_estimation: 69 # default is nseq/2 for folded SFS
pct_training: 0.67 # percentage of sites for training
nrand: 34 68 102 136 # number of random break points for each try (separated by white space)
project_dir: Amomum_fold # project directory
stairway_plot_dir: stairway_plot_es # directory to the stairway plot files
ninput: 200 # number of input files to be created for each estimation
#random_seed: 6
#output setting
mu: 1.4778e-9 # assumed mutation rate per site per generation
year_per_generation: 2 # assumed generation time (in years). The mu = 7.389e-10 * 2 = 1.4778e-9
#plot setting
plot_title: Amomum_fold # title of the plot
xrange: 0,0 # Time (1k year) range; format: xmin,xmax; "0,0" for default
yrange: 0,0 # Ne (1k individual) range; format: xmin,xmax; "0,0" for default
xspacing: 5 # X axis spacing
yspacing: 5 # Y axis spacing
fontsize: 12 # Font size
```

The blueprint file using SFS generated by vcf2sfs, assuming a generation time of 4 years:

```
#example blueprint file
#input setting
popid: Amomum # id of the population (no white space)
nseq: 138 # number of sequences
L: 12236258 # total number of observed nucleic sites, including polymorphic and monomorphic
whether_folded: true # whethr the SFS is folded (true or false)
SFS: 52 71 85 181 96 293 121 319 101 295 99 377 69 243 71 297 98 324 87 173 89 232 83 300 83 248 69 251 77 225 70 268 50 140 51 121 46 126 35 155 65 169 51 114 48 128 55 188 55 124 57 183 81 289 50 97 24 75 28 133 46 98 25 101 15 102 13 74 6 # snp frequency spectrum: number of singleton, number of doubleton, etc. (separated by white space)
#smallest_size_of_SFS_bin_used_for_estimation: 1 # default is 1; to ignore singletons, uncomment this line and change this number to 2
#largest_size_of_SFS_bin_used_for_estimation: 69 # default is nseq/2 for folded SFS
pct_training: 0.67 # percentage of sites for training
nrand: 34 68 102 136 # number of random break points for each try (separated by white space)
project_dir: Amomum_fold # project directory
stairway_plot_dir: stairway_plot_es # directory to the stairway plot files
ninput: 200 # number of input files to be created for each estimation
#random_seed: 6
#output setting
mu: 2.9556e-9 # assumed mutation rate per site per generation
year_per_generation: 4 # assumed generation time (in years). The mu = 7.389e-10 * 4 = 2.9556e-9
#plot setting
plot_title: Amomum_fold # title of the plot
xrange: 0,0 # Time (1k year) range; format: xmin,xmax; "0,0" for default
yrange: 0,0 # Ne (1k individual) range; format: xmin,xmax; "0,0" for default
xspacing: 5 # X axis spacing
yspacing: 5 # Y axis spacing
fontsize: 12 # Font size
```

The blueprint files used for Stairway Plot 2 analyses to produce Figure S2.

The blueprint file based on the SFS generated from group II individuals using easySFS,

assuming a generation time of 3 years.

```
#example blueprint file

#input setting

popid: Amomum # id of the population (no white space)

nseq: 44 # number of sequences

L: 11401907 # total number of observed nucleic sites, including polymorphic and monomorphic

whether_folded: true # whethr the SFS is folded (true or false)

SFS: 23.20939283575416 181.3277769025545 608.2353928158934 615.3292392460215 312.4540729305992 293.5614714153295 167.8143671867725 160.6824852366872 151.9329109042184 199.9159940469328 203.1105959664966 199.2997664239027 166.8120345092396 137.5012862443018 137.6536792592091 171.8942639431878 227.4127993473379 285.747658689921 268.5662117387836 229.1961361180318 166.3907437859197 74.94049784238426 # snp frequency spectrum: number of singleton, number of doubleton, etc. (separated by white space)

#smallest_size_of_SFS_bin_used_for_estimation: 1 # default is 1; to ignore singletons, uncomment this line and change this number to 2

#largest_size_of_SFS_bin_used_for_estimation: 22 # default is nseq/2 for folded SFS

pct_training: 0.67 # percentage of sites for training

nrand: 11 21 32 42 # number of random break points for each try (separated by white space)

project_dir: Amomum_fold # project directory

stairway_plot_dir: stairway_plot_es # directory to the stairway plot files

ninput: 200 # number of input files to be created for each estimation

#random_seed: 6

#output setting

mu: 2.2167e-9 # assumed mutation rate per site per generation

year_per_generation: 3 # assumed generation time (in years). The mu = 7.389e-10 * 3 = 2.2167e-9

#plot setting

plot_title: Amomum_fold # title of the plot

xrange: 0,0 # Time (1k year) range; format: xmin,xmax; "0,0" for default

yrange: 0,0 # Ne (1k individual) range; format: xmin,xmax; "0,0" for default

xspacing: 5 # X axis spacing

yspacing: 5 # Y axis spacing

fontsize: 12 # Font size
```

The blueprint file based on the SFS generated from group III individuals using easySFS, assuming a generation time of 3 years.

```
#example blueprint file
```

```
#input setting
popid: Amomum # id of the population (no white space)
nseq: 54 # number of sequences
L: 8435374 # total number of observed nucleic sites, including polymorphic and monomorphic
whether_folded: true # whethr the SFS is folded (true or false)
SFS: 4.223130114241016 44.04330817750256 207.0906634256965 270.0840252933715 165.5342764220935 165.7010000671132 104.583308229888 109.9384267827631 104.8137070275129 116.0326098971183 106.9327712087124 87.0513687083878 60.64801134380694 52.13830991081814 52.2616282369093 68.52178983953789 86.50131578633881 100.7898104503347 105.2127949892893 106.7738637007859 101.3001152906025 102.3247876679327 104.9009644822629 103.32176551371 101.0782639218116 91.75665560218853 44.29529969526026 # snp frequency spectrum: number of singleton, number of doubleton, etc. (separated by white space)
#smallest_size_of_SFS_bin_used_for_estimation: 1 # default is 1; to ignore singletons, uncomment this line and change this number to 2
#largest_size_of_SFS_bin_used_for_estimation: 27 # default is nseq/2 for folded SFS
pct_training: 0.67 # percentage of sites for training
nrand: 13 26 39 52 # number of random break points for each try (separated by white space)
project_dir: Amomum33_fold # project directory
stairway_plot_dir: stairway_plot_es # directory to the stairway plot files
ninput: 200 # number of input files to be created for each estimation
#random_seed: 6
#output setting
mu: 2.2167e-9 # assumed mutation rate per site per generation
year_per_generation: 3 # assumed generation time (in years). The mu = 7.389e-10 * 3 = 2.2167e-9
#plot setting
plot_title: Amomum_fold # title of the plot
xrange: 0,0 # Time (1k year) range; format: xmin,xmax; "0,0" for default
yrange: 0,0 # Ne (1k individual) range; format: xmin,xmax; "0,0" for default
xspacing: 5 # X axis spacing
yspacing: 5 # Y axis spacing
fontsize: 12 # Font size
```

The blueprint file based on the SFS generated from group II individuals using vcf2sfs, assuming a generation time of 3 years.

```
#example blueprint file
#input setting
popid: Amomum # id of the population (no white space)
```

```
nseq: 54 # number of sequences

L: 11401907 # total number of observed nucleic sites, including polymorphic and monomorphic

whether_folded: true # whethr the SFS is folded (true or false)

SFS: 0 0 388 859 159 482 108 229 73 151 55 309 78 309 59 168 50 177 52 272 76 519 92 205 42 65 7 # snp frequency spectrum: number of singleton, number of doubleton, etc. (separated by white space)

#smallest_size_of_SFS_bin_used_for_estimation: 1 # default is 1; to ignore singletons, uncomment this line and change this number to 2

#largest_size_of_SFS_bin_used_for_estimation: 27 # default is nseq/2 for folded SFS

pct_training: 0.67 # percentage of sites for training

nrand: 13 26 39 52 # number of random break points for each try (separated by white space)

project_dir: Amomum23_fold # project directory

stairway_plot_dir: stairway_plot_es # directory to the stairway plot files

ninput: 200 # number of input files to be created for each estimation

#random_seed: 6

#output setting

mu: 2.2167e-9 # assumed mutation rate per site per generation

year_per_generation: 3 # assumed generation time (in years). The mu = 7.389e-10 * 3 = 2.2167e-9

#plot setting

plot_title: Amomum_fold # title of the plot

xrange: 0,0 # Time (1k year) range; format: xmin,xmax; "0,0" for default

yrange: 0,0 # Ne (1k individual) range; format: xmin,xmax; "0,0" for default

xspacing: 5 # X axis spacing

yspacing: 5 # Y axis spacing

fontsize: 12 # Font size
```

The blueprint file based on the SFS generated from group III individuals using vcf2sfs, assuming a generation time of 3 years.

```
#example blueprint file

#input setting

popid: Amomum # id of the population (no white space)

nseq: 66 # number of sequences

L: 8435374 # total number of observed nucleic sites, including polymorphic and monomorphic

whether_folded: true # whethr the SFS is folded (true or false)

SFS: 0 0 73 354 60 279 39 164 35 119 38 200 27 119 24 61 30 75 27 95 29 144 30 162 23 135 32 152 22 147 13 60 0 # snp frequency spectrum: number of singleton, number
```

of doubleton, etc. (separated by white space)

#smallest_size_of_SFS_bin_used_for_estimation: 1 # default is 1; to ignore singletons, uncomment this line and change this number to 2

#largest_size_of_SFS_bin_used_for_estimation: 33 # default is nseq/2 for folded SFS

pct_training: 0.67 # percentage of sites for training

nrand: 16 32 48 64 # number of random break points for each try (separated by white space)

project_dir: Amomum33_fold # project directory

stairway_plot_dir: stairway_plot_es # directory to the stairway plot files

ninput: 200 # number of input files to be created for each estimation

#random_seed: 6

#output setting

mu: 2.2167e-9 # assumed mutation rate per site per generation

year_per_generation: 3 # assumed generation time (in years). The mu = 7.389e-10 * 3 = 2.2167e-9

#plot setting

plot_title: Amomum_fold # title of the plot

xrange: 0,0 # Time (1k year) range; format: xmin,xmax; "0,0" for default

yrange: 0,0 # Ne (1k individual) range; format: xmin,xmax; "0,0" for default

xspacing: 5 # X axis spacing

yspacing: 5 # Y axis spacing

fontsize: 12 # Font size
